# Downregulated expression of *S*_2_*-RNase* attenuates self-incompatibility in “Guiyou No. 1” pummelo

**DOI:** 10.1038/s41438-021-00634-8

**Published:** 2021-09-01

**Authors:** Jianbing Hu, Qiang Xu, Chenchen Liu, Binghao Liu, Chongling Deng, Chuanwu Chen, Zhuangmin Wei, Muhammad Husnain Ahmad, Kang Peng, Hao Wen, Xiangling Chen, Peng Chen, Robert M. Larkin, Junli Ye, Xiuxin Deng, Lijun Chai

**Affiliations:** 1 Key Laboratory of Horticultural Plant Biology, Ministry of Education, College of Horticulture and Forestry Sciences, Huazhong Agricultural University, Wuhan, 430070 People’s Republic of China; 2 Guangxi Engineering Research Center of Citrus Breeding and Culture, Guangxi Academy of Specialty Crops, Guilin, 541004 People’s Republic of China; 3 Guangxi Subtropical Crops Research Institute, Nanning, 530001 People’s Republic of China; 4 Horticulture Research Institute, Guangxi Academy of Agriculture Sciences, Nanning Investigation & Experiment Station of South Subtropical Fruit Trees, Ministry of Agriculture, Nanning, 530007 Guangxi People’s Republic of China; 5 Horticultural Institute, Hunan Academy of Agricultural Sciences, Changsha, 410125 People’s Republic of China

**Keywords:** Self incompatability, Gene regulation

## Abstract

Self-incompatibility (SI) substantially restricts the yield and quality of citrus. Therefore, breeding and analyzing self-compatible germplasm is of great theoretical and practical significance for citrus. Here, we focus on the mechanism of a self-compatibility mutation in ‘Guiyou No. 1’ pummelo (*Citrus maxima*), which is a spontaneous mutant of ‘Shatian’ pummelo (*Citrus maxima*, self-incompatibility). The rate of fruit set and the growth of pollen tubes in the pistil confirmed that a spontaneous mutation in the pistil is responsible for the self-compatibility of ‘Guiyou No. 1’. Segregation ratios of the *S* genotype in F_1_ progeny, expression analysis, and western blotting validated that the reduced levels of *S*_2_-*RNase* mRNA contribute to the loss of SI in ‘Guiyou No. 1’. Furthermore, we report a phased assembly of the ‘Guiyou No. 1’ pummelo genome and obtained two complete and well-annotated *S* haplotypes. Coupled with an analysis of SV variations, methylation levels, and gene expression, we identified a candidate gene (*CgHB40*), that may influence the regulation of the *S*_2_-*RNase* promoter. Our data provide evidence that a mutation that affects the pistil led to the loss of SI in ‘Guiyou No. 1’ by influencing a poorly understood mechanism that affects transcriptional regulation. This work significantly advances our understanding of the genetic basis of the SI system in citrus and provides information on the regulation of *S-RNase* genes.

## Introduction

Self-incompatibility (SI) is a reproductive strategy adopted by flowering plants to prevent inbreeding and promote outcrossing; therefore, it is a reproductive strategy that promotes genetic diversity^1^. The SI system has been classified into two types, sporophytic SI (SSI) and gametophytic SI (GSI), based on the genetic mechanism controlling the SI phenotype of the pollen^2,3^. A single polymorphic locus, the *S*-locus, contains two tightly linked *S* genes—the pollen and pistil *S* determinants—that confer the ability of individual plants to recognize and reject their own pollen^4,5^. Each SI system characterized to date relies on complex genetic mechanisms. In the *S-*RNase-based GSI system, the pistil *S* determinant encodes an exocrine protein (*S*-RNase) acting as a cytotoxic factor that inhibits the growth of pollen tubes by degrading RNAs^6^. Additionally, the *S*-locus F-box protein (SLF) serves as a pollen *S* determinant. SLF is a component of a Skp1-Cullin1-F-box (SCF) complex that promotes the growth of nonself pollen tubes by ubiquitinating and degrading nonself *S-RNases* in a 26S proteasome-dependent manner^7–10^. However, SI can limit reproduction, especially during agricultural production. This is a short-term disadvantage of SI that is expected to promote frequent transitions to SC^5^.

Loss of SI in plants is usually caused by mutations in key genes controlling self-incompatibility, mainly pistil *S* determinants^11,12^. Studies on *Petunia inflate*^13^, *Petunia axillaris*^14^, and *Solanum tuberosum*^15^ indicated that SI could be switched to SC by directly attenuating or abolishing the expression of the *S-RNase* gene. In addition, epigenetic modifications in the 5′-flanking region of *S*_*f*_*-RNase* can significantly affect its expression and lead to the loss of SI in almond^16^. In brief, the *S*-haplotype-specific rejection of pollen requires the maintenance of a high expression level of the *S-RNase* gene^8^. Additionally, the ribonuclease activity of *S-RNases* is essential for the rejection of pollen^8,17^. The reduction in *S*-RNase enzyme activity contributed to the loss of SI in Chinese pear (*Pyrus bretschneideri* Rehd.)^18,19^ and *Petunia inflata*^20^.

Citrus is one of the most widely cultivated and economically important fruit crops worldwide. Many citrus accessions are self-incompatible, such as Hassaku (*Citrus hassaku* hort. ex Tanaka)^21^, Hyuganatsu (*Citrus tamurana* hort. ex Tanaka)^22^, ‘Wuzishatangju’ (*Citrus reticulata* Blanco)^23^, ‘Shatian’ pummelo (*Citrus grandis* Osbeck)^24^, Clementine mandarin (*Citrus clementina* Hort. ex Tan.)^25^, ‘W. Murcott Afourer’ mandarin (*Citrus reticulata*)^26^, ‘Xiangshui’ lemon [*C. limon* (L.) Burm. F.]^27^, and ‘Kagzi Kalan’ lemon (*Citrus limon*)^28^. Our previous studies have demonstrated that in citrus, *S*-RNase is a female determinant of GSI^29–33^. The full-length cDNA clones of *S-RNase* in citrus contain coding regions ranging from 660 to 699 bp that encode highly polymorphic proteins with predicted molecular masses between 25.1 and 26.8 kDa, and alkaline isoelectric points ranging from 7.69 to 9.44. Each amino acid sequence contains five conserved domains and two hypervariable regions (Supplementary Fig. 2). The single intron in each gene is located between the two hypervariable regions^29^. In citrus, a number of studies have been performed on the transition from SI to SC. For instance, ‘Wuzishatangju’ (*C. reticulata* Blanco, SI) was derived from a bud sport of ‘Shatangju’ (*C. reticulata* Blanco, SC)^23,34^. ‘Monreal’ (*Citrus clementina* Hort. ex Tan., SC) was derived from a self-compatible mutant of ‘Comune’ (*Citrus clementina* Hort. ex Tan., SI) that occurred naturally^35^. ‘Nishiuchi Konatsu’ (*Citrus tamurana* hort. ex Tanaka, SC) was derived from a bud sport variation of ‘Hyuganatsu’ (*Citrus tamurana* hort. ex Tanaka, SI)^22^. Nevertheless, mechanistic studies on the mutations that influence self-compatibility and self-incompatibility in citrus are rare, with the exception of *Sm*^29^.

**Fig. 1:**
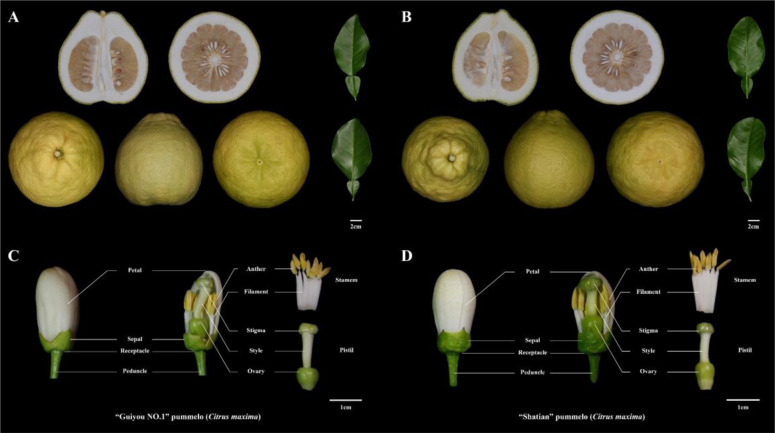
Images of pummelo fruits, leaves, and floral organs. **A**, **C** “Guiyou No. 1” pummelo (*Citrus maxima*); **B**, **D** “Shatian” pummelo (*Citrus maxima*)

In this article, we investigated the robust self-compatibility of ‘Guiyou No.1’ pummelo (*Citrus maxima*, hereafter referred to as GY), which is a spontaneous mutant of ‘Shatian’ pummelo (*Citrus maxima*, referred to as ST) (Fig. 1). Data from our pollen tube growth assays and the segregation ratios of the *S* genotype in the F_1_ progeny indicate that the downregulated expression of *S*_2_-*RNase* contributes to the loss of SI in ‘GY’. Moreover, we used a procedure that included an analysis of SV variation, methylation levels, and gene expression data to identify a candidate gene (*CgHB40*) that potentially contributes to the regulation of the promoter from *S*_2_*-RNase*. This work may fill a gap in our knowledge of the upstream regulation of *S-RNase* genes in citrus.

## Materials and methods

### Plant materials and sample extraction

The plant materials used in this study were from two cultivars of pummelo (*Citrus maxima)* that were collected from Rong County, Yulin City, Guangxi Province, China. One cultivar is the self-incompatible ‘Shatian’ pummelo (hereafter abbreviated as ST). The other cultivar is the ‘Guiyou No. 1’ pummelo (hereafter abbreviated as GY), which was derived from a bud sport of ‘ST’ (Fig. 1 and Supplementary Table 1). Fresh leaves and floral organs of ‘ST’ and ‘GY’ were collected, frozen immediately in liquid nitrogen, and then stored at −80 °C for further analysis. Fresh leaf tissues were used for genomic sequencing.

### Pollination assay

To test whether ‘GY’ pummelo is an SC variety, self- and cross-pollinations were carried out with ‘ST’ and ‘GY’. Nearly opened flowers from the two cultivars were collected, and pollen was obtained by manually separating and drying the anthers at 28 °C for 12 h. One d before anthesis, floral buds were emasculated and hand-pollinated using a fine paint brush and covered with a paper pollination bag. Approximately 60–70 flowers were pollinated on 3–4 individual plants for each type of cross. Aniline blue staining was performed as described by Liang et al.^36^.

### RT-PCR analysis

Total RNA was extracted with RNAiso Plus (TaKaRa) according to the manufacturer’s instructions. cDNA was synthesized with HiScript II Q RT SuperMix for qPCR (+gDNA wiper) (Vazyme Biotech) according to the supplier’s protocol. For RT-PCR, 100 μg of cDNA was added to each reaction system. The PCR program for all isoforms was as follows: 95 °C for 3 min, 30× (95 °C for 15 s, 57 °C for 15 s, and 72 °C for 30 s), and 72 °C for 5 min. Products were separated on 1.5% TAE-agarose gels and photographed. All samples were run in triplicate, and there were two separate runs for each plate. The pertinent primers are listed in Supplementary Table 4.

### Expression and purification of recombinant *S*-RNase

The coding sequence from *S*_1_ and *S*_2_*-RNase* without the signal peptide was cloned into pGEX-6p-1 to create the GST-S-RNase fusion protein in an *E. coli* strain overexpressing C43 (DE3) (WeiDi Biotech) as previously described^29^. GST fusion proteins were overexpressed in *E. coli* cultures when the OD_600_ was 0.6–0.8 by adding 0.2 mM IPTG. Cells were harvested after 16 h at 20 °C.

The recombinant *S*_1_-RNase and *S*_2_-RNase proteins were purified with GST SefinoseTM Resin (Sangon Biotech) as recommended by the manufacturer. For the RNase activity analysis, the solvent was exchanged with a solution containing 0.1 M sodium succinate and 0.01 M KCl at pH 6.0 using an Amicon centrifugation device (molecular mass cutoff, 10 kDa) (Millipore) at 4 °C. Protein concentrations of the samples were determined with an Omni-Easy^TM^ Instant BCA Protein Assay Kit, and the samples were stored at −80 °C.

### *S*-RNase activity analysis

RNase activity assays were performed as previously described with slight modifications^37^. Torula yeast RNA (10 mg ml^−1^, Sangon Biotech) dissolved in 0.1 M sodium succinate, and 0.01 M KCl, pH 6.0, was incubated at 55 °C for 10 min before being used as the substrate. Then, 125 μL of reaction buffer containing 0.1 M sodium succinate (pH 6.0), 0.01 M KCl, and 10 μg protein was also incubated at 55 °C for 10 min before the addition of 100 μL of RNA solution. The reaction was carried out for 30 min at 55 °C and stopped by the addition of 25 μL of ice-cold trichloroacetic acid (50%). Following a 15 min incubation on ice, the solution was centrifuged at 12,000 × *g* for 10 min. The supernatant was diluted 25-fold with ultrapure water. The absorbance was measured at 260 nm with an Infinite M200 Pro NanoQuant (TECAN). Specific activity was defined as the A260 units released from torula yeast RNA into an acid-soluble form from 1.0 mg of the protein in 1 min at 55 °C. The data are presented as the mean values ± SE from three independent biological replicates.

### Protein extraction from styles and western blot analysis

Styles were excised from the pistils of flowers that were collected 1 d before anthesis. Total protein was extracted from styles using the Plant Protein Extraction Kit (epizyme) according to the manufacturer’s protocol. Approximately 5−7 μg of total protein was analyzed with 10% SDS gels and then by immunoblotting using PVDF membranes. Polyclonal anti-*S*_1_-RNase antibodies (1:1,000 dilution) and anti-*S*_2_-RNase antibodies (1:1,000 dilution) were generated against the *S*_1_-RNase-GST and *S*_*2*_-RNase-GST fusion proteins in rabbits, respectively (FriendBio Technology, Wuhan, Hubei, China). These antibodies were used to detect *S*_1_-RNase and *S*_2_-RNase proteins, respectively, using a goat anti-rabbit IgG-horseradish peroxidase-conjugated secondary antibody (Abbkine, 1:5,000 dilution).

### Genomic and transcriptomic sequencing

The sequencing of single molecules was performed on the PacBio Sequel II platform (Pacific Biosciences, USA) by Beijing Biomarker Technologies Co. Ltd. (Beijing, China). A SMRTbell library with a size of 30 kb was constructed for genomic sequencing following the standard protocol provided by Pacific Biosciences. To survey the genome information, a 300 bp library was constructed to generate short reads (150 bp paired-end) using the BGISEQ-500 platform (BGI-Shenzhen, China).

After total RNA extraction and DNase I treatment, magnetic beads with Oligo (dT) were used to isolate mRNA. The mRNA was mixed with fragmentation buffer and fragmented into short fragments. cDNA was synthesized using mRNA fragments as templates. Short fragments were purified and resolved with EB buffer for end repair and single nucleotide A (adenine) addition. The short fragments were connected with adapters. Suitable fragments were selected and used as templates for PCR amplification. During the QC steps, the Agilent 2100 Bioanalyzer and the ABI StepOnePlus Real-Time PCR System were used for the quantification of the sample library. Subsequently, the library was sequenced using the BGISEQ-500 platform (BGI-Shenzhen, China).

### Genome assembly and *S* haplotype reconstruction

A total of 220.94 Gb CLR reads were assembled using Canu (v2.1.1)^38^ with the following parameters: -- pacbio (minReadLength = 2000, minOverlapLength = 500, corOutCoverage = 150, corMinCoverage =2 “batOptions = -dg 3 -db 3 -dr 1 -ca 500 -cp 50”, corrected error rate= 0.035). Canu generated two assemblies composed of contigs and unitigs. The unitig assembly consisted of the contigs that split at any alternative path in the assembly graph. The contig assembly had longer continuity but more chimeric fragments as revealed by the genetic mapping analysis. The unitigs were polished iteratively using two rounds of Pilon with ~12 Gb of WGS BGISEQ data to generate the draft genome GYgv1 (CNA0019670). The sequences of the *S*_1_ and *S*_2_ haplotypes were identified from the GYgv1 assembled genome using the *S-RNase* gene. The GY-*S*_1_ and GY-*S*_2_ loci from ‘GY’ pummelo were obtained using the conserved sequences at each end of the *S* loci^29^. Gene predictions and annotations of GY-*S*_1_ and GY-*S*_2_ loci were made using FGENESH^39^ and nonredundant protein sequence (nr) databases^40^. Genes containing an F-box domain and an F-box-associated motif were designated SLFs. The ST-*S*_1_ and ST-*S*_2_ loci were obtained from publicly available databases (see Supplementary Tables 3, 5, and 6 for more details). The syntenic regions of GY-*S*_1_ and GY-*S*_2_ were identified using the all-vs-all BLASTP (e-value = 1e−5) method with a threshold value of 0.95 identity.

### Whole-sequence alignments and structural variation analysis

Pairwise alignments of all *S* loci were performed using NUCmer from MUMmer (v.4.0.0)^41^ with the --prefix option and HWB-*S*_6_ loci as a reference^42^. The ‘ST’ *S*_1_ and *S*_2_ loci were obtained from publicly available databases (see Supplementary Table 3 for more details). Whole-sequence synteny analysis between the ‘GY’ pummelo *S* haplotypes was carried out with NUCmer and the --prefix option using the HWB-*S*_6_ haplotype as a reference. Structural variants (SVs; >50 bp) were called using show-diff and show-snps, respectively. The SNPs were called using show-snps from MUMmer (v.4.0.0)^41^ and the -1 filter.

### Transcriptome analysis

To gain insight into the mechanism responsible for self-compatibility in ‘GY’ pummelo, a comprehensive transcriptome analysis of the anther and style of ‘ST’ and ‘GY’ pummelo was performed to identify differentially expressed genes (DEGs) in different tissues. Anther and style samples were collected 1 d before anthesis. The reproducibility of transcriptome samples was assessed using a Spearman rank correlation. Expression levels were calculated as transcripts per kilobase per million mapped reads (TPM). The DEGs from the transcriptome data were analyzed using the R package DEseq2^43^. The significant DEGs were determined using a threshold of log2FoldChange > 1 with an adjusted *P*-value < 0.01. Furthermore, the GO (Gene Ontology) database and the KEGG (Kyoto Encyclopedia of Genes and Genomes) pathway database were utilized to identify enriched biological functions and metabolic pathways.

### Whole-genome bisulfite sequencing (WGBS) and analysis

Genomic DNA was purified from the style tissue 1 d before anthesis using the CTAB method. For the construction of paired-end BS-seq libraries using the NEXTflex Bisulfite-Seq kit (Bioo Scientific), genomic DNA was converted to approximately 300 bp fragments using an ultrasonic homogenizer, end-repaired, and 3′ adenylated. The products were ligated with methylated adapters and subjected to size selection using the MiniElute PCR Purification Kit (QIAGEN). Bisulfite treatment was carried out using the EZ DNA Methylation-Gold kit (Zymo Research) according to the manufacturer’s instructions. The constructed libraries were validated using the Agilent Technologies 2100 bioanalyzer and paired-end sequenced with 150 bp reads on the BGISEQ-500 platform (BGI-Shenzhen, China). For data analysis, adapters and low-quality reads were removed. Clean reads were mapped to the GYgv1 genome using Bismark (v0.22.2)^44^, allowing up to three mismatches. For the identification of differentially methylated regions (DMRs), fractional DNA methylation levels were calculated using 200 bp sliding windows with 50 bp step sizes in the *S-RNase* regions of the ‘ST’ and ‘GY’ genomes. Only cytosines from both ‘ST’ and ‘GY’ that were covered by at least four reads were considered and counted. The methylation level of individual cytosines was calculated as the ratio of mC to the total cytosines [mC/(mC+un-mC)].

### Yeast one‐hybrid assay

The Y1H assay was conducted as previously described^45^. As bait, the promoter sequences of *S2-RNase* (2340 bp) were cloned into pAbAi vectors and used to transform the Y1HGold yeast strain. Then, the bait strains were transformed with the pGADT7‐Cg1g003830.1 fusion plasmid. The cotransformed yeast cells were cultured on SD/‐Leu agar plates with or without aureobasidin A (AbA) and incubated at 30 °C for 2−3 d.

### Dual-luciferase (LUC) assay

For LUC, an effector vector was constructed by cloning the full-length *CgHB40* CDS into the pGreenII 62-SK vector, whereas reporter vectors were generated by fusing the p*S*_2_-F2 promoter fragment (735 bp, the second part of *S*_2_-*RNase* promoter) into pGreenII 0800-LUC. Effectors and reporters were cotransformed into *Nicotiana benthamiana* leaves and the activity of LUC and REN was measured using the Dual-Luciferase^Ⓡ^. Reporter Assay System (Promega, USA). Promoter activity was determined by the ratio of LUC/REN. LUC was imaged with the Plant Fluorescent Imaging System in Vivo after the application of luciferin on the abaxial leaf surface.

## Results

### A pistil-side mutation confers self-compatibility to ‘GY’ pummelo

‘GY’ is a spontaneous mutant of ‘ST’ (*S*_*1*_*S*_*2*_). The *S* genotype was determined to be *S*_1_*S*_2_ using specific primer pairs (Supplementary Table 1). To test the stability of the self-compatibility in ‘GY’, the fruit set rate and the average number of seeds per fruit after the self-pollination and cross-pollination of ‘GY’ and ‘ST’ were determined (Table 1). The different percentages of fruit set from the self-pollination of ‘ST’ and ‘GY’, 2.5 and 30.8%, respectively, indicated that ‘GY’ possesses self-compatibility. The fruit produced by the F_1_ population that was derived from the ‘GY’ × ‘GY’ cross contained tenfold more seeds than the fruit produced by the F_1_ population that was derived from the ‘ST’ × ‘ST’ cross. The germination rate of the F_1_ seeds derived from the ‘GY’ × ‘GY’ cross was nearly 100%. In contrast, the seeds derived from the self-pollination of ‘ST’ barely germinated (Table 2). There was another significant difference in the outcome of the reciprocal crosses between ‘GY’ and ‘ST’. The fruit set ratio of the cross-pollinated ‘GY’♀ × ‘ST’♂ was remarkably higher than the fruit set ratio of the cross-pollinated ‘ST’♀ × ‘GY’♂, although the number of seeds per fruit from these two cross-pollination experiments was not significantly different (Table 1).

**Table 1:** Comparison of fruit set from four pollination experiments

Pollination	No. of pollinated flowers	No. of fruit	Fruit set ratio (%)	No. of total seeds	No. of seeds per fruit
GY♀ × GY♂	26	8	30.8	1109	138.6
GY♀ × ST♂	37	5	13.5	743	148.6
ST♀ × ST♂	40	1	2.5	11	11
ST♀ × GY♂	27	1	3.7	116	116

The data in Table 1 were collected in 2020.

**Table 2:** Segregation of *S* genotypes in F_1_ progeny

Genetic cross	No. of progeny	Possible genotypes^a^	Observed ratio^b^	Expected ratio^c^	*χ*^2^ value	*P* value
GY (*S*_1_*S*_2_) × GY (*S*_1_*S*_2_)	530	*S*_1_*S*_1_:*S*_1_*S*_2_:*S*_2_*S*_2_	0:274:256	0:1:1	0.6113208	0.434290956
				1:2:1	247.91698	1.4639E−54 ^**^
GY (*S*_1_*S*_2_) × ST (*S*_1_*S*_2_)	309	*S*_1_*S*_1_:*S*_1_*S*_2_:*S*_2_*S*_2_	0:150:159	0:1:1	0.2621359	0.60865653
				1:2:1	163.8932	2.57658E−36 ^**^
ST (*S*_1_*S*_2_) × ST (*S*_1_*S*_2_)	0	*S*_1_*S*_1_:*S*_1_*S*_2_:*S*_2_*S*_2_				
ST (*S*_1_*S*_2_) × GY (*S*_1_*S*_2_)	81	*S*_1_*S*_1_:*S*_1_*S*_2_:*S*_2_*S*_2_	27:35:19	1:2:1	3.0740741	0.215017247
				1:1:1	4.7407407	0.09344611

Segregation analysis of *S* haplotypes of F_1_ progeny of pummelo accessions from self- and cross-pollination experiments analyzed using PCR (Fig. 3D–F).

^a^The observed genotypes are underlined.

^b^The S-genotype ratios observed in all of the progeny.

^c^The upper segregation ratio is that expected from a single genotype mutation model of the GSI system, while the lower segregation ratio is that expected from simple Mendelian inheritance. The pollinations that used pistils from ‘GY’ produced results that were consistent with the single genotype mutation model, with a nonsignificant chi-square value for this prediction and a highly significant difference (**P < 0.001) for the lower segregation ratio. These data provide compelling evidence that the mutation that promotes self-compatibility in ‘GY’ occurs on the pistil side.

**Fig. 2:**
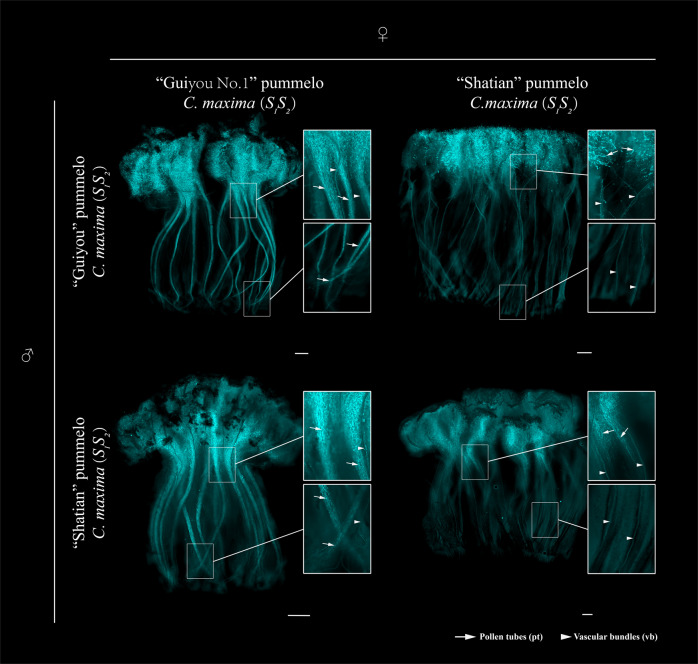
Fluorescence images of pollen tubes in pistils after different pollinations. Representative fluorescence images (five of each pollination combination) of aniline blue-stained pistils at stage -1 DBA (1 d before anthesis) 5 d after pollination are shown. The accession names and their respective *S*- genotypes are indicated to the left and at the top. Scale bars = 1 mm. Pollen tubes (pt) are indicated with arrows. Vascular bundles (vb) are indicated with arrowheads

When ‘ST’ and ‘GY’ were used as sources of pollen for ‘ST’ pistils at 1 d before anthesis, virtually no pollen tube growth was observed at the middle and base of the styles at 5 d post pollination. In contrast, when ‘GY’ was used as the pollen receptor for pollen from ‘ST’ and ‘GY’, pollen tubes were observed at the bottom of the styles (Fig. 2). Therefore, ‘GY’ developed an anther-side SI phenotype but not a pistil-side SI phenotype. These observations are consistent with the fruit-set rate of ‘GY’ after the self- and cross-pollination experiments (Table 1). These data indicate that ‘GY’ is stably self-compatible and that the loss of SI was probably caused by a pistil-side mutation.

### Downregulated expression of *S*_*2*_*-RNase* promotes SC in ‘GY’ pummelo

**Fig. 3:**
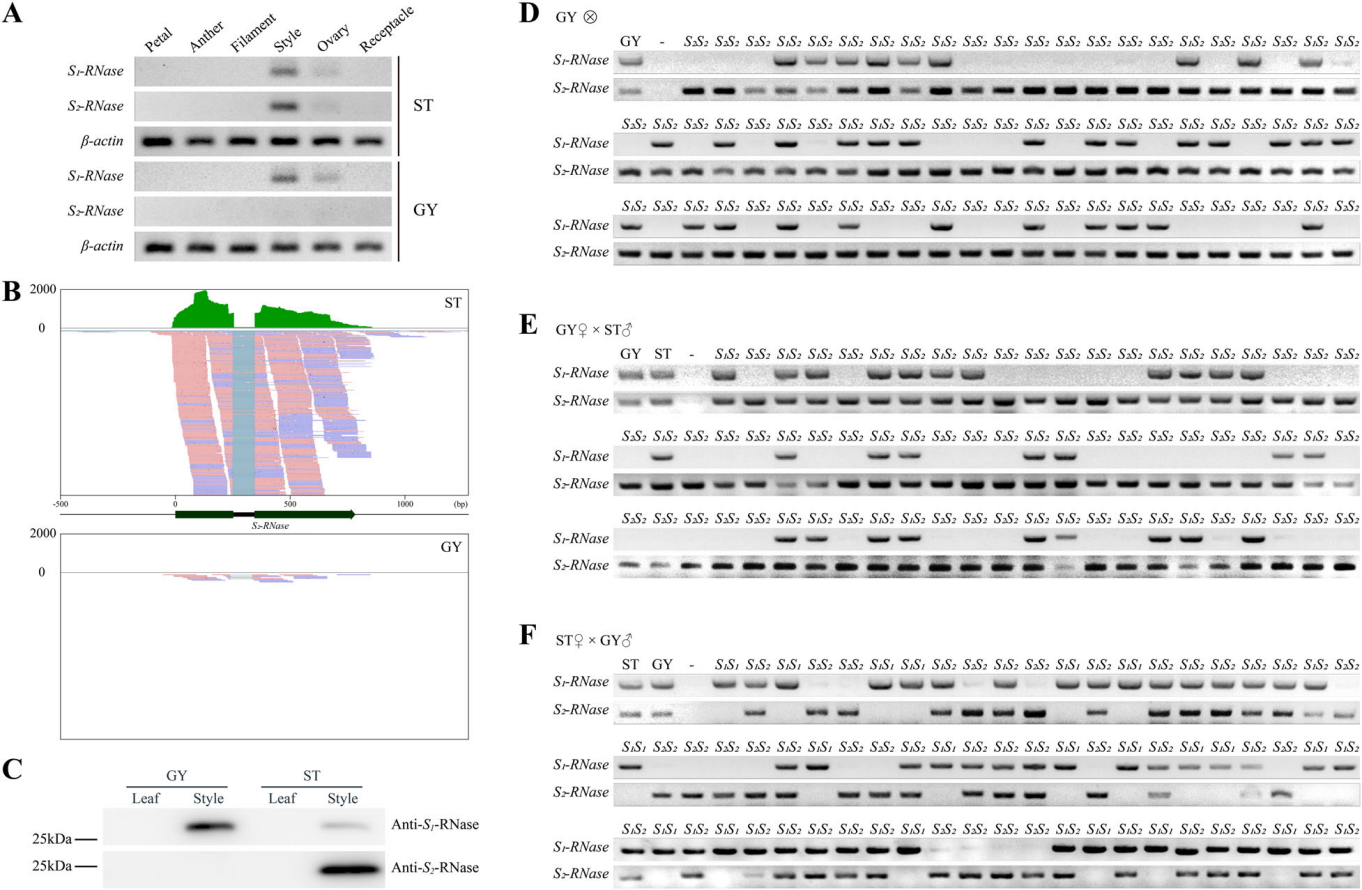
Attenuated expression of *S*_2_*-RNase* leads to SC in ‘GY’ pummelo. **A** Expression of *S*_1_*-RNase* and *S*_*2*_*-RNase* in different tissues from ‘ST’ and ‘GY’. Expression was quantified using RT–PCR. *S*_2_*-RNase* is expressed at much lower levels in the styles of ‘GY’ relative to the styles of ‘ST’, and it is expressed in a tissue-specific manner, being present only in the ovary and style tissues of pistils. The expression levels of *S*_1_*-RNase* in the styles from ‘GY’ and ‘ST’ were similar. **B** Sequence read clusters from the *S*_2_*-RNase* gene. The sequence read clusters are from the RNA-Seq data generated from the styles of ‘GY’ and ‘ST’ pummelo and are shown in the Integrative Genomics Viewer. The green bars depict the number of reads mapped to the assembled reference genome for ‘GY’. There were significantly more reads mapped to *S*_2_*-RNase* in the ‘ST’ styles than in the ‘GY’ styles. A partial alignment of the RNA mapping data is shown below, with pink and blue representing the different read strands. **C** Tissue-specific expression of the *S*_1_-RNase and *S*_2_-RNase proteins in the pistils of ‘ST’ and ‘GY’ pummelo. On an immunoblot, the antibodies raised against recombinant *S*_1_-RNase and *S*_2_-RNase cross-react with a protein of approximately 25 kDa in extracts prepared from the mature pistils of ‘ST’ pummelo. Only *S*_1_-RNase was detected in the pistil of ‘GY’. No *S*_2_-RNase protein was detected in extracts prepared from the pistils of ‘GY’ pummelo. The *S*_1_-RNase and *S*_2_-RNase antisera did not cross-react with a protein in leaf tissue. Segregation of *S-RNase*s with the *S* locus in F_1_ progeny from different cross combinations (**D** GY × GY cross; **E** GY × ST cross; **F** ST × GY cross; *S*_1_*S*_2_ × *S*_1_*S*_2_). Genotyping of the progeny from each cross using PCR with *S*_1_- and *S*_2_*-RNase* primers showed that the parental pistils carried the same *S*_1_- and *S*_2_-*RNase* genes. Pairs of amplified *S-RNase* sequences in three different combinations (*S*_1_*S*_1_, *S*_1_*S*_2_*,* or *S*_2_*S*_2_) were amplified from the 70 progeny from each type of cross

To identify the pistil-side mutation responsible for the transition from SI to SC in ‘GY’, the segregation ratios of the *S* genotypes in the F_1_ progeny from the four pollination combinations were analyzed using chi-square tests. None of the 11 seeds generated by the self-pollinated ‘ST’ germinated. In contrast, the *S* haplotypes of the progeny from the self-pollinated ‘GY’ segregated into two classes (*S*_1_*S*_2_:*S*_2_*S*_2_) at a ratio of 274:256 (Table 2; Fig. 3D). This ratio is consistent with an abnormity linked to the *S*_*2*_ locus (1:1, *χ*^2^ = 0.6113, *P* > 0.05; Table 2). Furthermore, 309 individual F_1_ progeny derived from the ‘GY’ × ‘ST’ cross exhibited either the *S*_1_*S*_2_ (150 plants) or *S*_2_*S*_2_ (159 plants) genotypes in the expected 1:1 ratio (*χ*^2^ = 0.2621, *P* = 0.6087; Table 2; Fig. 3E). Nevertheless, the *S*_1_*S*_1_ genotype was absent from the F_1_ progeny, although it was a possible outcome of crosses that utilized pistils from ‘GY’ (Fig. 3D, E). These data demonstrate that functional *S*_1_-RNase is present in the pistils of ‘GY’, resulting in a partial ability to reject pollen from ‘ST’ and ‘GY’. In contrast, the *S*_2_ genotype existed in all of the individual F_1_ progeny produced by the pistils from ‘GY’ (Fig. 3D, E). These data indicate that dysfunction of *S*_2_-RNase may disrupt SI in ‘GY’.

Therefore, we analyzed the expression of *S-RNase* genes in floral tissues. Not surprisingly, *S*_1_*-RNase* and *S*_2_*-RNase* were expressed normally in the pistils of ‘ST’. In contrast, only the expression of *S*_1_*-RNase* was detected in the pistils of ‘GY’ (Fig. 3A, B; Supplementary Fig. 4). Subsequently, the abundance of *S*_2_-RNase proteins in the pistils of ‘GY’ was investigated. For this purpose, crude protein extracts from the style and leaf tissues of ‘GY’ and ‘ST’ were prepared. The results from the western blot analysis were consistent with the results from RT-PCR in that although the *S*_1_-RNase protein was detected in the styles of both ‘GY’ and ‘ST’, the *S*_2_-RNase protein was present only in the styles of ‘ST’ (Fig. 3C). Moreover, the analysis of RNase activity indicated that the activity of *S*_2_-RNase was higher than the activity of *S*_1_-RNase (Supplementary Fig. 3A). These data provide evidence that both the *S*_2_-RNase and *S*_1_-RNase proteins are functional *S*-determinants and that the attenuated expression of *S*_2_*-RNase* induced a transition from SI to SC in ‘GY’.

### Diversity in *S* loci does not attenuate the expression of *S*_2_*-RNase*

To better understand the attenuated expression of *S*_2_*-RNase* in the style of ‘GY’, we cloned the *S*_2_*-RNase* gene and 2 kb of sequence upstream of the *S*_2_*-RNase* gene. We compared this sequence to the *S*_2_*-RNase* gene from ‘ST’ and found no differences in the 2 kb 5′ flanking sequence in ‘GY’ and ‘ST’ (i.e., no SNPs or other types of polymorphisms were detected) (Supplementary Fig. 2). Some structural variants (SVs) of the *S*-locus can be responsible for the loss of self-incompatibility^46–49^. Thus, we sequenced and assembled the genome of ‘GY’. A total of 3,969,910,472 subreads and 220.94 Gb (≈ 552X, Table 3 and Supplementary Fig. 1) of clean data from PacBio long reads were generated from sequencing one whole flow cell. The genome was based on single-molecule real-time (SMRT) DNA sequencing and de novo assembled with Canu^38^, which produces a partially phased diploid genome. Finally, a total of 1,429 contigs (≥100 bp) were assembled into 566.29 Mb with a contig N50 of 1.11 Mb. We used a BUSCO^50^ analysis to evaluate the integrity of the genome assembly. Among the 2,326 conserved genes, 2,252 orthologs (98.9%) of these genes were completely aligned to the assembled genome. Only 19 orthologs (0.9%) were not detected in the genome (Table 3).

**Table 3:** Summary statistics for assembly of the ‘GY’ pummelo genome sequence

Sequencing	*Platform*	*Library size*
	PacBio Sequel II	30 kb
	*Clean data (Gb)*	*Coverage (X)*
	220.94	552
Genome assembly	*Assembly*	*Primary assembly*
	Assembly length (bp)	587,752,981
	Number of sequences	1,429
	Average length (bp)	411,303
	Maximum length (bp)	18,341,039
	Minimum length (bp)	9,822
	N50 length (bp)	1,159,493
	GC content (%)	34.6%
BUSCO	*Types*	*Percentage (%)*
	Complete BUSCOs (C)	98.9
	Complete single-copy BUSCOs (S)	23.6
	Complete and duplicated BUSCOs (D)	75.3
	Fragmented BUSCOs (F)	0.2
	Missing BUSCOs (M)	0.9

**Fig. 4:**
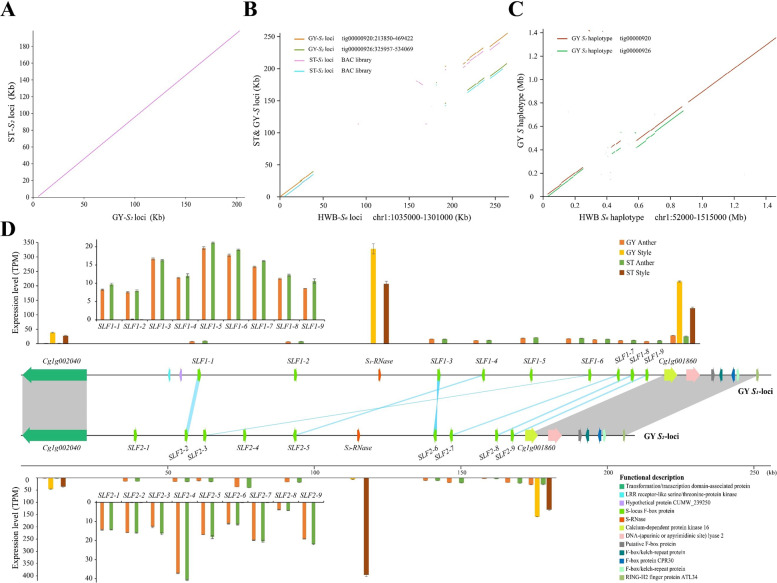
Structural variant analysis of *S* loci. **A** Whole-sequence alignments of the *S*-locus region in *S*_2_ haplotypes against the corresponding loci in ‘ST’. **B** Whole-sequence alignments of the *S*-locus region in *S*_1_ and *S*_2_ haplotypes against the HWB *S*_6_-locus reference genome. The ST *S*_1_ and *S*_2_ loci were obtained from publicly available databases (see Supplementary Table 3 for more details). **C** Whole-sequence synteny analysis of the ‘GY’ pummelo *S*_*1*_ and *S*_*2*_ haplotypes against the HWB S6 haplotype reference. The GY-*S*_1_ haplotype and the GY-*S*_2_ haplotype were obtained from the phased assembled genome. Haplotype phasing was carried out using CANU (v2.1.1). **D** Schematic representations of the *S* loci in the ‘GY’ assembled genome. The expression levels of genes at the *S*_1_ and *S*_2_ loci in the style and anther tissues of ‘ST’ and ‘GY’ pummelo were based on three biological replicates. TPM values for the genes in the *S*_1_ and *S*_2_ loci were calculated with the reads mapped to the assembled reference genome. Error bars indicate mean values ± SE, *n* = 3. Gene synteny for the *S*_1_ and *S*_2_ loci is shown in the middle row of the panel. Gray lines indicate syntenic regions at the end of the *S* loci. Blue lines indicate syntenic sequences in the intergenic regions of the *S*_1_ and *S*_2_ loci. Colored boxes represent different genes associated with the *S*_1_ and *S*_2_ loci. Orange boxes represent *S-RNase*. Green boxes represent *SLFs* (see Supplementary Tables 5–7 for more details)

The *S* haplotypes were identified by the presence of the *S-RNase* genes on the tig00000920 and tig00000926 contigs of the GYgv1 reference genome. The GY-*S*_*1*_ and GY-*S*_2_ loci from the ‘GY’ pummelo were obtained using the conserved sequences at each end of the *S* loci. In our previous work^29^, the sequences of the ST-*S* loci were obtained from a BAC library (Supplementary Table 3). All of the *S* haplotypes were aligned to the HWB *C. maxima S*_6_ haplotype to assess structural differences and to identify *S* loci-specific features. The sequences of the *S* loci were highly polymorphic (Fig. 4B). Whole-sequence alignment analysis showed that the sequences flanking both ends—approximately 37 kb on the left flank and approximately 43 kb on the right flank—of the *S*_1_, *S*_2,_ and *S*_6_ loci were well conserved. The *S* loci that determine the self-incompatibility of citrus were located in the middle of this approximately 150 kb highly polymorphic region (Fig. 4B; Supplementary Fig. 3C). We manually annotated the genes in the region of the *S* loci using the GYgv1 genome sequence and RNA-Seq analysis. Compared with the ST-*S*_2_ and GY-*S*_2_ loci, we found that the *S*_2_ locus from ‘GY’ was completely identical to that of ‘ST’ and that there was no SV variation between these two *S* loci (Fig. 4A, C; Supplementary Fig. 3B). Collinearity analysis also showed that the structure and gene content of the region flanking the GY-*S* locus was highly conserved and that the 7 *SLF* genes (*SLF1-1*, *SLF1-3*, *SLF1-4*, *SLF1-6*, *SLF1-7*, *SLF1-8*, and *SLF1-9* and *SLF2-2*, *SLF2-6*, *SLF2-5*, *SLF2-3*, *SLF2-7*, *SLF2-8*, and *SLF2-9*) from each *S* locus were highly collinear (Fig. 4D and Supplementary Tables 5–7), which was consistent with the previous studies^29^.

An analysis of the mean transcript levels from the *S*_1_ and *S*_2_ loci in the style and anther tissues of ‘ST’ and ‘GY’ pummelo indicated no significant difference between the expression of the *SLF* and *S*_1_*-RNase* genes in ‘ST’ relative to ‘GY’ in each tissue with the exception of the expression of the *S*_2_*-RNase* gene in the style of ‘GY’. The expression of the *S*_2_*-RNase* gene was significantly attenuated in the ‘GY’ style relative to the ‘ST’ style (Fig. 4D). Sequence and expression analysis indicated that variation in the *S* loci sequences is not responsible for the attenuated expression of the *S*_2_*-RNase* gene in the styles of ‘GY’.

Epigenetic modifications make a major contribution to gene silencing^16,51,52^. To test whether DNA methylation might attenuate the expression of *S*_2_*-RNase*, we used WGBS to profile DNA methylation in the styles from both ‘ST’ and ‘GY’. Two biological replicates from each genotype were sequenced. For each sample, at least 120 M paired-end reads (read length = 150 bp) were produced. Approximately 87% of the reads were mapped to the GYgv1 reference genome using Bismark^44^. These data covered >99.9% of the genome. The average depth of coverage at cytosines was 16.60-fold and 16.58-fold in the styles of ‘ST’ and ‘GY’, respectively (Supplementary Tables 8 and 9).

In the styles of ‘ST’ and ‘GY’ pummelo, ~20.2 and 19.3% of the cytosines are methylated, which were defined using a binomial test as described by Zhong^52^. The average CpG, CHG, and CHH methylation levels were 38, 35, and 26%, respectively. The largest proportion of mCs was in the CHH context. The methylation rates of ‘ST’ and ‘GY’ in the CHH context were 7.3 and 7%, respectively. The second-highest proportion of mCs was in the CHG context. The numbers of mCs in the CHG context were 142.5 and 138.1 million in ‘ST’ and ‘GY’, respectively. The corresponding methylation percentages were higher than 35%. mCs in the CpG context were methylated at the highest average levels of methylation, reaching 7.8 and 7.4% in ‘ST’ and ‘GY’, respectively (Supplementary Table 8).

**Fig. 5:**
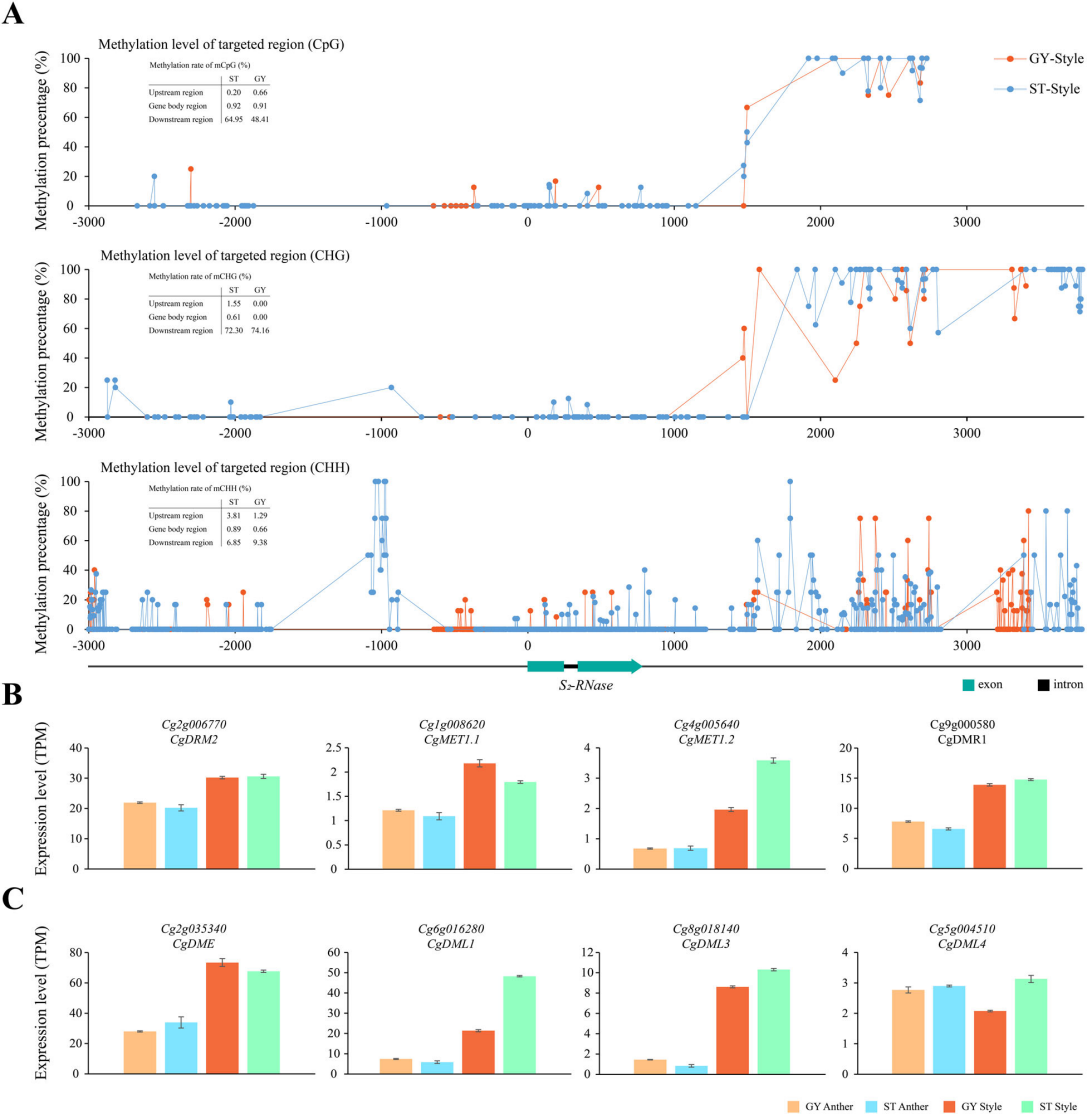
Methylation status of *S*_2_*-RNase* in the styles of ‘ST’ and ‘GY’. **A** Methylation levels in the *S*_2_*-RNase* genes in the styles of ‘ST’ and ‘GY’. Methylation levels in the CpG (top row), CHG (middle row), and CHH (bottom row) were quantified in 3 kb long 5′-flanking regions, exons, introns and 3 kb long 3′-flanking regions. The percentage of total mCs is the number of mCs/total number of Cs. **B**, **C** Expression of DNA methyltransferase and DNA demethylase genes in the anthers and styles of ‘ST’ and ‘GY’. Transcript levels of DNA methyltransferase (B) and demethylase (C) genes in the anther and style tissues. Error bars indicate mean values ± SE, *n* = 3

We calculated the average DNA methylation rates for the *S-RNase* regions and found that DNA methylation gradually increased in the 3′ flanking regions of the *S*_2_*-RNase* gene and that the largest proportion of mCs was in the CHH context. The corresponding methylation rates were 6.85 and 9.38%, respectively. The second-highest proportion of mCs was in the CHG context, which had the highest methylation rates. The corresponding methylation rates were 72.3 and 74.2%, respectively (Fig. 5A). However, the methylation rates of the promoter and gene body were significantly lower than those of the downstream region. The average methylation rates of the promoter and *S*_2_*-RNase* gene body in the CpG, CHG, and CHH contexts were consistent with the methylation rates that were observed in the styles of ‘ST’ and ‘GY’. Moreover, the average methylation levels in the different contexts were similar in the regions containing *S*_1_*-RNase* and *S*_2_*-RNase* in ‘ST’ and ‘GY’ (Fig. 5A and Supplementary Fig. 5C). Thus, the similar levels and patterns of methylation in the regions containing *S*_1_*-RNase* and *S*_2_*-RNase* may not strongly contribute to the attenuated expression of *S*_2_*-RNase* in the styles of ‘ST’ and ‘GY’.

In the HWB *C. maxima* genome^42^, we identified seven genes encoding methyltransferase orthologs using BLASTP. A phylogenetic tree was constructed using the maximum-likelihood method (Supplementary Fig. 5A, B). Three of these seven methyltransferase genes (*Cg2g001280*, *CgUng003970,* and *Cg3g008940*) were barely expressed [transcripts per kilobase of exon model per million mapped reads (TPM) < 1] in style tissue (Fig. 5B and Supplementary Fig. 5D). Among the other four genes, there was no significant difference in expression levels in ‘GY’ relative to those in ‘ST’. This result is consistent with the similar levels of methylation in these two cultivars (Fig. 5B). Regarding DNA demethylase genes, we identified four *AtROS1* orthologs, including *Cg2g035340* (*CgDME*), *Cg6g016280* (*CgDML1*), *Cg8g018140* (*CgDML3*), and *Cg5g004510* (*CgDML4*). Among the four pummelo DNA demethylase genes, *Cg5g004510* (TPM < 3) was expressed at lower levels in styles than the other three genes (TPM > 10). The expression of *Cg6g01628* in the styles of ‘GY’ was significantly lower than that in the styles of ‘ST’. The relative expression levels of the three other genes were not significantly different in the style and anther tissues of ‘ST’ and ‘GY’ (Fig. 5C). The methyltransferase and DNA demethylase genes were expressed at higher levels in the styles than in the anthers (Fig. 5B, C). The results provide evidence that variation in the levels of methylation in the styles of ‘ST’ and ‘GY’ may not be caused by changes in the expression levels of any of the genes that encode DNA methyltransferases and demethylases. In conclusion, we did not observe a significant correlation between the average levels of mC in the regions containing the *S-RNase* loci and the attenuated expression of *S*_2_*-RNase* in the styles of ‘GY’.

### Differential gene expression in pistils may attenuate the expression of *S*_2_*-RNase*

**Fig. 6:**
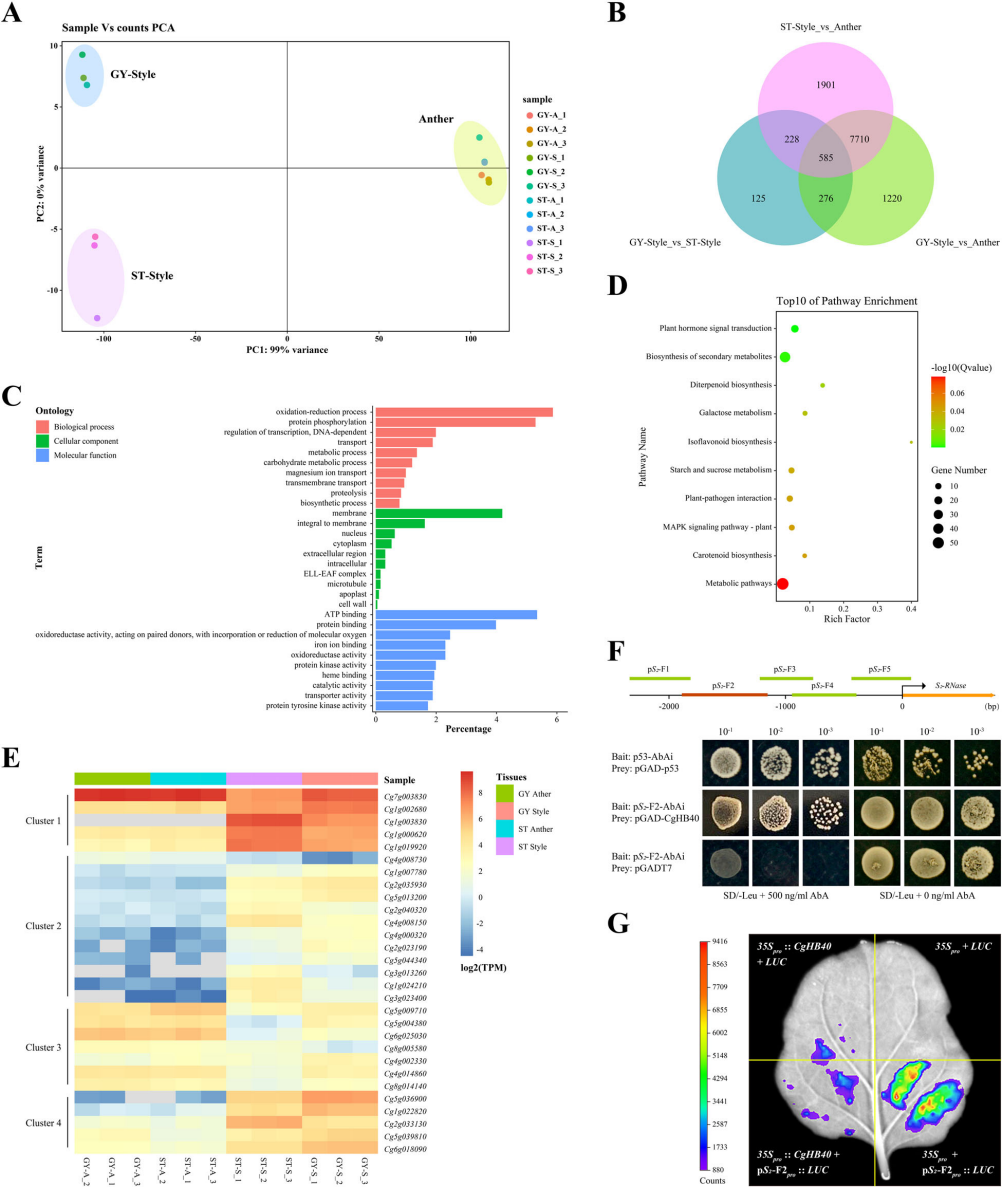
Differential expression analysis of the anther and style tissues from ‘ST’ and ‘GY’ pummelo. **A** Principal component analysis (PCA) of RNA-Seq data from floral tissues. The data indicate consistency among the three biological replicates. In the key (right), the samples are named with the following formula: A-B_C. A indicates the accession code (Supplementary Table 1), B indicates the tissue (“*S*” indicates style tissues and “*A*” indicates anther tissues), C indicates a particular biological replicate (i.e., 1, 2, or 3). **B** DEGs in the anther and style tissues of ‘ST’ and ‘GY’ pummelo. The numbers of DEGs are indicated. **C** Ten representative GO terms enriched in 585 DEGs. BP biological process; CC cellular component; MF molecular function. **D** Top ten significantly enriched KEGG pathways in the 585 DEGs. **E** Heatmaps showing the expression of 29 differentially expressed genes encoding transcription factors. **F** Interactions between CgHB40 (Cg1g003830) and the promoter of *S*_2_*-RNase*. Interactions between CgHB40 and the promoter of *S*_2_*-RNase* were demonstrated using the yeast one-hybrid system. p53-AbAi+pGAD and p*S*_*2*_-F2+pGADT7 were expressed in yeast as positive and negative controls, respectively. **G** Transient expression assays showing that CgHB40 inhibits the expression of p*S*_2_-F2_pro_:: *LUC*. Representative images of *Nicotiana benthamiana* leaves 72 h after infiltration are shown

The mechanism that attenuates the expression of *S*_2_*-RNase* and thus contributes to the loss of SI in ‘GY’ pummelo was explored using a transcriptome profiling approach that utilized RNA-based sequencing (RNA-Seq) of the floral tissues in ‘ST’ and ‘GY’. Approximately 40 million clean reads per sample were obtained (Supplementary Table 2). Analysis of the Spearman rank correlation demonstrated consistency among the three biological replicates. Additionally, the correlation coefficient of each sample was higher than 0.98, indicating good reproducibility (Supplementary Fig. 6). Thus, these RNA-Seq data were used for further analysis. Although principal component analysis (PCA) indicated consistent expression profiles in the anthers of ‘ST’ and ‘GY’, the transcriptomes from the styles did not cluster together (Fig. 6A), which indicates that the transcriptomes from the styles were significantly different. We assumed that the different transcriptome profiles in the ‘ST’ and ‘GY’ styles may contribute to the different expression levels of *S*_2_*-RNase* mRNA.

To test this hypothesis, 9791 (style vs. anther in ‘GY’, from 1 d before anthesis (-1 DBA)), 10424 (style vs. anther in ‘ST’, -1 DBA), and 1214 (style in ‘GY’ vs. style in ‘ST’, -1 DBA) differentially expressed genes (DEGs) were identified using a ≥2 fold change and a false discovery rate (FDR) ≤0.05 as the criteria for differential expression (Fig. 6B). The 585 DEGs could explain the transition from SI to SC in ‘GY’ pummelo (Supplementary Table 10). To understand the main biological functions associated with the DEGs, Gene Ontology (GO) enrichment analysis was performed against the GO database using *P* ≤ 0.05 as the threshold. The main biological functions associated with the 585 DEGs were categorized into 90 biological processes (BP), 21 cellular components (CC), and 161 molecular functions (MF) (Supplementary Table 11). In the BP category, the top three terms were ‘oxidation−reduction process’ (GO:0055114, 112, 5.87%), ‘protein phosphorylation’ (GO:0006468, 101, 5.29%), and ‘regulation of transcription, DNA-dependent’ (GO: 0006355, 38, 1.99%) (Fig. 6C). To gain insight into the metabolic pathways associated with the differentially expressed genes, the genes were annotated with a Kyoto Encyclopedia of Genes and Genomes (KEGG) pathway enrichment analysis against the KEGG database. Although the main metabolic processes associated with the 585 DEGs were classified into 62 enriched pathways, only nine of these pathways were significantly enriched (*q* ≤ 0.05). The top three significantly enriched pathways were ‘plant hormone signal transduction’ (ko04075), ‘biosynthesis of secondary metabolites’ (ko01110), and ‘diterpenoid biosynthesis’ (ko00904). These significantly enriched pathways mainly belonged to environmental information processing and metabolism in the KEGG_A class (Fig. 6D, Supplementary Table 12). GO and KEGG analyses demonstrated that the regulation of transcription biological processes and signal transduction pathways may participate in the regulation of *S*_2_*-RNase* expression.

Therefore, we tried to demonstrate a rationale for the differential expression of *S*_2_-RNase in ‘GY’ relative to ‘ST’ from the perspective of transcriptional regulation. We identified 29 transcription factor genes among the 585 DEGs. Based on the expression profiles in anther and style tissues, 29 TF genes clustered into four groups (Fig. 6E). Genes in clusters 1 and 4 were mainly expressed in styles with particular tissue specificities. Importantly, the expression levels of genes in ‘ST’ and ‘GY’ were significantly different (i.e., fold change > 2). Therefore, yeast one-hybrid (Y1H) assays were performed with these ten genes to test whether they could bind to the promoter of *S*_2_*-RNase* in yeast. We found that only Cg1g003830 bound to the promoter from *S*_2_*-RNase* (Fig. 6F and Supplementary Fig. 7). Thus, *Cg1g003830* (hereafter referred to as *CgHB40*) was selected for further study. A 2340 bp segment of the *S*_*2*_*-RNase* promoter was used as bait in Y1H assays. CgHB40 was expressed as a fusion to the GAL4 activation domain. The full-length *S*_*2*_*-RNase* promoter and several promoter fragments (p*S*_2_-F1, p*S*_2_-F3, p*S*_2_-F4, and p*S*_2_-F5) appeared to strongly self-activate such that the strains containing the full-length *S*_2_*-RNase* promoter and particular promoter fragments grew well on the selective medium. Indeed, 900 ng/mL AbA could not inhibit the growth of these strains. However, although pGADT7 + p*S*_2_-F2 did not grow on the selective medium (SD/-Leu + 500 ng/mL AbA), pGAD-CgHB40 + p*S*_2_-F2 did grow on the selective medium, indicating an interaction between CgHB40 and the p*S*_2_-F2 promoter (Fig. 6F).

Transient luciferase (LUC) imaging assays were also performed to verify the binding of CgHB40 to the *S*_2_-*RNase* promoter in vivo. In these assays, the *CaMV35S* promoter was used to express CgHB40 (i.e., the effector). The plasmid (p*S*_2_*-*F2_pro_::*Luc*) containing the promoter of *S*_2_-*RNase* fused to the reporter gene luciferase, was combined with *35S*_pro_::*CgHB40* and coinfiltrated into tobacco leaves. A Strong luminescence signal was detected in the coexpressed regions of *35S*_pro_ + p*S*_*2*_*-*F2_pro_::*Luc*, but much weaker and no luminescence signals were detected in *35S*_pro_::*CgHB40* + p*S*_*2*_*-*F2_pro_::*Luc* and the negative controls, respectively. Taken together, these results indicate that CgHB40 can regulate the activity of the p*S*_*2*_*-*F2 promoter (Fig. 6G). Based on these data, CgHB40 is a strong candidate for a transcription factor that regulates the transcription of *S*_2_*-RNase* in styles. More experiments are required to determine whether CgHB40 contributes to the loss of SI in ‘GY’.

## Discussion

The transition from an outcrossing to a selfing mating system is one of the most prevalent evolutionary transitions in flowering plants^5,53^. Hence, the number of studies on the transition from SI to SC has increased. Our understanding of the mechanisms responsible for SI has improved^15,18^. In this study, we used genetic and pollination assays to demonstrate that the loss of SI in ‘GY’ was due to a pistil-side mutation. The fruit setting rate of self-pollinated ‘GY’ was remarkably higher than the fruit setting rate of self-pollinated ‘ST’ (30.8 and 2.5%, respectively). Moreover, the results from the pollination experiments were consistent with the average seed numbers from the fruit (138.6 and 11, respectively) (Table 1). Nevertheless, the self-incompatibility in the styles of ‘ST’ should have prevented the self-pollinated ‘ST’ from producing fruit with seeds (Fig. 2). However, we obtained 11 and 116 individuals from one fruit each of the ‘ST’ self-cross and ‘ST’ ◊ ‘GY’ cross combinations, respectively. The rates of fruit set were 2.5 and 3.7%, respectively (Table 1). These unexpected results are consistent with those of previous studies^54,55^ and may be explained by the environment or by the accidental pollination of one or two flowers in the budding stage. Nonetheless, our work suggests that a pistil-side mutation is responsible for self-compatibility in ‘GY’ pummelo.

The mechanisms responsible for the loss of SI can be grouped into three categories that involve gene duplications and mutations in the *S-RNase*, *SLF,* or non-*S* determinant genes^56,57^. Not surprisingly, the results of our expression analysis and the segregation ratios of the *S* genotype in the F_1_ progeny demonstrated that the attenuated expression of *S*_*2*_*-RNase* contributed to the loss of SI in ‘GY’ pummelo (Fig. 3A–C; Supplementary Fig. 4). Therefore, to better understand the mechanism that attenuates the expression of *S*_2_*-RNase* in the styles of ‘GY’, we prepared a phased assembly of the ‘GY’ genome (GYgv1) and obtained two complete and well-annotated *S* haplotypes. To date, research related to pistil dysfunction in the realm of SI has mainly focused on sequence variation that results in the reduction of RNase activity and its expression^17,18^. However, a comparison of the structural variation in ‘GY’ relative to ‘ST’ did not explain the loss of SI in ‘GY’. No duplications, inversions, deletions, or translocations were observed between the same *S*-loci of ‘ST’ and ‘GY’. Moreover, we did not observe SNPs in 5 kb regions on the 5′ and 3′ flanks of the *S-RNase* genes (Fig. 4A; Supplementary Fig. 3B). Thus, we were able to completely exclude the possibility that sequence variations in the *S*_2_ locus attenuated the expression of *S*_2_*-RNase* in ‘GY’.

The methylation of cytosine residues was proposed to suppress gene expression^58^. The methylation of several cytosine residues in a 4700 bp fragment in the 5′ upstream region of *S*_*f*_*-RNase* was strongly associated with the inactivation of the *S*_*f*_ allele in all self-compatible varieties of almond^16^. Here, we performed WGBS for ‘ST’ and ‘GY’ styles and calculated the average DNA methylation rates of the *S-RNase* regions. Despite epigenetic changes downstream of the *S-RNase* genes, the average methylation levels in different contexts were distributed similarly in the *S*_1_*-RNase* and *S*_2_*-RNase* regions of ‘ST’ and ‘GY’ (Fig. 5A and Supplementary Fig. 5C). The absence of significant differences in DNA methylation rates in the regions containing the *S*_1_ and *S*_2_ loci of ‘GY’ and ‘ST’ provides evidence that an unknown modifier gene may affect the expression of *S*_2_*-RNase* genes. Obviously, these observations conflict with a previous study on methylated cytosine residues. The researchers argued that the farthest site (M4) was correlated with the silencing of the *S*_*f*_*-RNase* gene in almond^16^. Currently, this conclusion seems unreliable because this study did not completely exclude the influence of different nucleotide sequences, and the analysis of methylation was limited to the CpG context and did not cover the entire *S*_*f*_*-RNase* region. However, we cannot conclude that methylation does not contribute to the regulation of *S-RNase* genes. Indeed, changes in methylation levels were detected in the noncoding region of *S-RNase* genes. Nonetheless, much more work is required to test this idea.

Many years ago, similar research was performed in *Petunia axillaris*. The author proposed a model that included a modifier locus (*MDF*) that is unlinked to the *S* locus and that could specifically affect the expression of *S*_13_*-RNase*^14^. *MDF* is probably a transcription factor that specifically regulates the expression of a particular *S-RNase* gene. Nevertheless, due to the polymorphic nature of *S* loci, little is known about the regulation of the *S-RNase* genes. Here, by performing a transcriptome analysis, a candidate gene (*CgHB40*) was identified that may be involved in the transcriptional regulation of *S*_2_*-RNase* genes in styles. Additional experiments are required to test whether the candidate contributes to the loss of SI in ‘GY’. This work significantly advances our understanding of the genetic basis of the breakdown of the SI system in citrus and provides the information necessary to regulate the expression of the *S-RNase* genes.

## Supplementary information

Supplementary materials

Supplementary Figure 1

Supplementary Figure 2

Supplementary Figure 3

Supplementary Figure 4

Supplementary Figure 5

Supplementary Figure 6

Supplementary Figure 7

Supplementary Table 10

Supplementary Table 11

Supplementary Table 12

## Data Availability

All of the sequence data and the genome assembly have been deposited in the CNGB Sequence Archive (CNSA)^59^ of the China National GeneBank DataBase (CNGBdb)^60^ with accession numbers CNP0001704, CNP0001705, and CNP0001706 (https://db.cngb.org/).

## References

[1] Yamamoto M, Nishio T (2014). Commonalities and differences between *Brassica* and *Arabidopsis* self-incompatibility. Hortic. Res..

[2] Fujii S, Kubo K, Takayama S (2016). Non-self- and self-recognition models in plant self-incompatibility. Nat. Plants.

[3] Tao R, Iezzoni AF (2010). The S-RNase-based gametophytic self-incompatibility system in *Prunus* exhibits distinct genetic and molecular features. Sci. Hortic..

[4] Matsumoto D, Tao R (2016). Distinct self-recognition in the *Prunus* S-RNase-based gametophytic self-incompatibility system. Hortic. J..

[5] Goldberg EE (2010). Species selection maintains self-incompatibility. Science.

[6] Wu L (2018). Use of domain-swapping to identify candidate amino acids involved in differential interactions between two allelic variants of type-1 S-locus F-box protein and S3-RNase in *Petunia inflata*. Plant Cell Physiol..

[7] Guo H, Halitschke R, Wielsch N, Gase K, Baldwin IT (2019). Mate selection in self-compatible wild tobacco results from coordinated variation in homologous self-incompatibility genes. Curr. biol..

[8] Takayama S, Isogai A (2005). Self-incompatibility in plants. Annu. Rev. Plant Biol..

[9] Hua Z, Kao TH (2006). Identification and characterization of components of a putative *Petunia S*-locus F-box-containing E3 ligase complex involved in S-RNase-based self-incompatibility. Plant Cell Online.

[10] Entani T (2014). Ubiquitin-proteasome-mediated degradation of S-RNase in a solanaceous cross-compatibility reaction. Plant J..

[11] Li W (2016). Molecular and genetic characterization of a self-compatible apple cultivar, ‘CAU-1’. Plant Sci..

[12] Baldwin SJ, Schoen DJ (2017). Genetic variation for pseudo-self-compatibility in self-incompatible populations of *Leavenworthia alabamica* (Brassicaceae). New Phytol..

[13] Lee HS, Huang S, Kao T (1994). S proteins control rejection of incompatible pollen in *Petunia inflata*. Nature.

[14] Tsukamoto T (2003). Breakdown of self-incompatibility in a natural population of *Petunia axillaris* caused by a modifier locus that suppresses the expression of an S-RNase gene. Sex. Plant Reprod..

[15] Ye M (2018). Generation of self-compatible diploid potato by knockout of *S-RNase*. Nat. Plants.

[16] Fernandez I Marti A, Gradziel TM, Socias I Company R (2014). Methylation of the *S*_*f*_ locus in almond is associated with *S*-RNase loss of function. Plant Mol. Biol..

[17] Huang S, Lee HS, Karunanandaa B, Kao TH (1994). Ribonuclease activity of *Petunia inflata* S proteins is essential for rejection of self-pollen. Plant Cell..

[18] Li Y (2020). A mutation near the active site of S-RNase causes self-compatibility in S-RNase-based self-incompatible plants. Plant Mol. Biol..

[19] Li MF, Li XF, Han Zh H, Shu HR, Li T (2009). Molecular analysis of two Chinese pear (*Pyrus bretschneideri* Rehd.) spontaneous self-compatible mutants, Yan Zhuang and Jin Zhui. Plant Biol. (Stuttg.)..

[20] McCubbin AG, Chung YY, Kao T (1997). A Mutant S_3_ RNase of *Petunia inflata* lacking RNase activity has an allele-specific dominant negative effect on self-incompatibility interactions. Plant Cell..

[21] Yamashita K, Tanimoto S (1985). Studies on self-incompatibility of Hassaku (*Citrus hassaku* hort. ex Tanaka). J. Jpn Soc. Hortic. Sci..

[22] Honsho C (2009). Reproductive characteristics for self-compatibility and seedlessness in ‘Nishiuchi Konatsu’, a bud mutation of hyuganatsu (*Citrus tamurana* hort. ex Tanaka). Hortscience.

[23] Ye W (2009). Seedless mechanism of a new mandarin cultivar ‘Wuzishatangju’ (*Citrus reticulata* Blanco). Plant Sci..

[24] Chai L, Ge X, Biswas MK, Xu Q, Deng X (2010). Self-sterility in the mutant ‘Zigui shatian’ pummelo (*Citrus grandis* Osbeck) is due to abnormal post-zygotic embryo development and not self-incompatibility. Plant Cell Tiss. Org..

[25] Caruso M (2012). Comparative transcriptome analysis of stylar canal cells identifies novel candidate genes implicated in the self-incompatibility response of *Citrus Clementina*. BMC Plant Biol..

[26] Gambetta G (2013). Self-incompatibility, parthenocarpy and reduction of seed presence in ‘Afourer’ mandarin. Sci. Hortic..

[27] Zhang S (2015). Characterization of the ‘Xiangshui’ lemon transcriptome by de novo assembly to discover genes associated with self-incompatibility. Mol. Genet. Genomics.

[28] Kakade V, Dubey AK, Sharma RM, Malik SK (2017). Gametophytic self-incompatibility causes seedlessness in ‘Kagzi Kalan’ lemon (*Citrus limon*). J. Hortic. Sci. Biotech..

[29] Liang M (2020). Evolution of self-compatibility by a mutant *S*_*m*_-RNase in citrus. Nat. Plants.

[30] Miao HX, Qin YH, Teixeira da Silva JA, Ye ZX, Hu GB (2011). Cloning and expression analysis of S-RNase homologous gene in *Citrus reticulata* Blanco cv. Wuzishatangju. Plant Sci..

[31] Liang M (2016). Genome-wide identification and functional analysis of S-RNase involved in the self-incompatibility of citrus. Mol. Genet. Genomics.

[32] Ma Y, Li Q, Hu G, Qin Y (2017). Comparative transcriptional survey between self-incompatibility and self-compatibility in *Citrus reticulata* Blanco. Gene.

[33] Lin W (2019). Two genes (*ClS1* and *ClF-box*) involved the self-incompatibility of “Xiangshui” lemon (*Citrus limon* (L.) Burm. f.). Plant Mol. Biol. Rep..

[34] Miao H, Ye Z, Hu G, Qin Y (2015). Comparative transcript profiling of gene expression between self-incompatible and self-compatible mandarins by suppression subtractive hybridization and cDNA microarray. Mol. Breed..

[35] Distefano G, Caruso M, La Malfa S, Gentile A, Tribulato E (2009). Histological and molecular analysis of pollen-pistil interaction in clementine. Plant Cell Rep..

[36] Liang M (2017). Genome-wide identification and functional analysis of S-RNase involved in the self-incompatibility of citrus. Mol. Genet Genomics.

[37] Brown PH, Ho TH (1986). Barley aleurone layers secrete a nuclease in response to gibberellic acid: purification and partial characterization of the associated ribonuclease, deoxyribonuclease, and 3’-nucleotidase activities. Plant Physiol..

[38] Koren S (2017). Canu: scalable and accurate long-read assembly via adaptive *k*-mer weighting and repeat separation. Genome Res..

[39] Solovyev V, Kosarev P, Seledsov I, Vorobyev D (2006). Automatic annotation of eukaryotic genes, pseudogenes and promoters. Genome Biol..

[40] Pruitt KD, Tatusova T, Maglott DR (2005). NCBI Reference Sequence (RefSeq): a curated non-redundant sequence database of genomes, transcripts, and proteins. Nucleic Acids Res..

[41] Marcais G (2018). MUMmer4: a fast and versatile genome alignment system. PLoS Comput Biol..

[42] Wang X (2017). Genomic analyses of primitive, wild, and cultivated citrus provide insights into asexual reproduction. Nat. Genet..

[43] Love MI, Huber W, Anders S (2014). Moderated estimation of fold change and dispersion for RNA-seq data with DESeq2. Genome Biol..

[44] Krueger F, Andrews SR (2011). Bismark: a flexible aligner and methylation caller for Bisulfite-Seq applications. Bioinformatics.

[45] Duan Z (2020). The *Brassica napus* GATA transcription factor BnA5.ZML1 is a stigma compatibility factor. J. Integr. Plant Biol..

[46] Huang SX (2008). Competitive interaction between two functional *S*-haplotypes confer self-compatibility on tetraploid Chinese cherry (*Prunus pseudocerasus* Lindl. CV. Nanjing Chuisi). Plant Cell Rep..

[47] Okada K (2008). Deletion of a 236 kb region around *S*_4_*-RNase* in a stylar-part mutant *S*_*4*_^*sm*^-haplotype of Japanese pear. Plant Mol. Biol..

[48] Yamane H, Ikeda K, Hauck NR, Iezzoni AF, Tao R (2003). Self-incompatibility (*S*) locus region of the mutated *S*^6^-haplotype of sour cherry (*Prunus cerasus*) contains a functional pollen *S* allele and a non-functional pistil *S* allele. J. Exp. Bot..

[49] Tsuchimatsu T (2017). Patterns of polymorphism at the self-incompatibility locus in 1,083 *Arabidopsis thaliana* genomes. Mol. Biol. Evol..

[50] Seppey, M., Manni, M. & Zdobnov, E. M. In *Gene Prediction: Methods and Protocols* (ed. Martin Kollmar) 227−245 (Springer, 2019).

[51] Zou L (2020). Gene body demethylation increases expression and is associated with self-pruning during grape genome duplication. Hortic. Res..

[52] Huang H (2019). Global increase in DNA methylation during orange fruit development and ripening. Proc. Natl Acad. Sci. USA..

[53] Nasrallah JB (2017). Plant mating systems: self-incompatibility and evolutionary transitions to self-fertility in the mustard family. Curr. Opin. Genet Dev..

[54] Suyama T (2013). Overcoming self-incompatibility by bud pollination and hot-water treatment in interspecific hybrids of *Hydrangea*. Horticultural Res. (Japan).

[55] Wakana A, Ngo BX, Fukudome I, Kajiwara K (2004). Estimation of the degree of self-incompatibility reaction during flower bud development and production of self-fertilized seeds by bud pollination in self-incompatible Citrus cultivars. J. Fac. Agr. Kyushu U..

[56] Stone JL (2002). Molecular mechanisms underlying the breakdown of gametophytic self-incompatibility. Q. Rev. Biol..

[57] McClure B, Cruz-Garcia F, Romero C (2011). Compatibility and incompatibility in S-RNase-based systems. Ann. Bot..

[58] Jones PA, Takai D (2001). The role of DNA methylation in mammalian epigenetics. Science.

[59] Guo, X. et al. CNSA: a data repository for archiving omics data. *Database* **2020**, baaa055 (2020). 10.1093/database/baaa055 PMC7377928 32705130

[60] Chen FZ (2020). CNGBdb: China National GeneBank DataBase. Yi Chuan.

