# Supplementary material for: Downregulated expression of S2-RNase attenuates self-incompatibility in “Guiyou No. 1” pummelo
Source: Hortic Res. 2021 Sep 1;8:199. doi: 10.1038/s41438-021-00634-8 (PMC8408199; doi:10.1038/s41438-021-00634-8)
Supplement: Supplementary file 1 — Supplementary materials [file 41438_2021_634_MOESM1_ESM.docx]

# Supplemental Information for：

# Downregulated expression of *S_2_-RNase* attenuates Self-incompatibility in “Guiyou NO.1” pummelo

Jianbing Hu ^1^, Qiang Xu ^1^, Chenchen Liu ^1^, Binghao Liu ^2^, Chongling Deng ^2^, Chuanwu Chen ^2^, Zhuangmin Wei ^3^, Muhammad Husnain Ahmad ^1^, Kang Peng ^1^, Hao Wen ^1^, Xiangling Chen ^4^, Peng Chen ^5^, Robert M. Larkin ^1^, Junli Ye ^1^, Xiuxin Deng ^1^, Lijun Chai ^1,^*

^1^ Key Laboratory of Horticultural Plant Biology, Ministry of Education, College of Horticulture and Forestry Sciences, Huazhong Agricultural University, Wuhan 430070, P. R. China;

^2^ Guangxi Engineering Research Center of Citrus Breeding and Culture, Guangxi Academy of Specialty Crops, Guilin 541004, P. R. China;

^3^ Guangxi Subtropical Crops Research Institute, Nanning 530001, P. R. China;

^4^ Horticulture Research Institute, Guangxi Academy of Agriculture Sciences, Nanning Investigation & Experiment Station of South Subtropical Fruit Trees, Ministry of Agriculture, Nanning 530007, Guangxi, P. R. China;

^5^ Horticultural Institute, Hunan Academy of Agricultural Sciences, Changsha, 410125, China;

*Corresponding author:

E-mail: chailijun@mail.hzau.edu.cn

**Supplementary Figures**

**
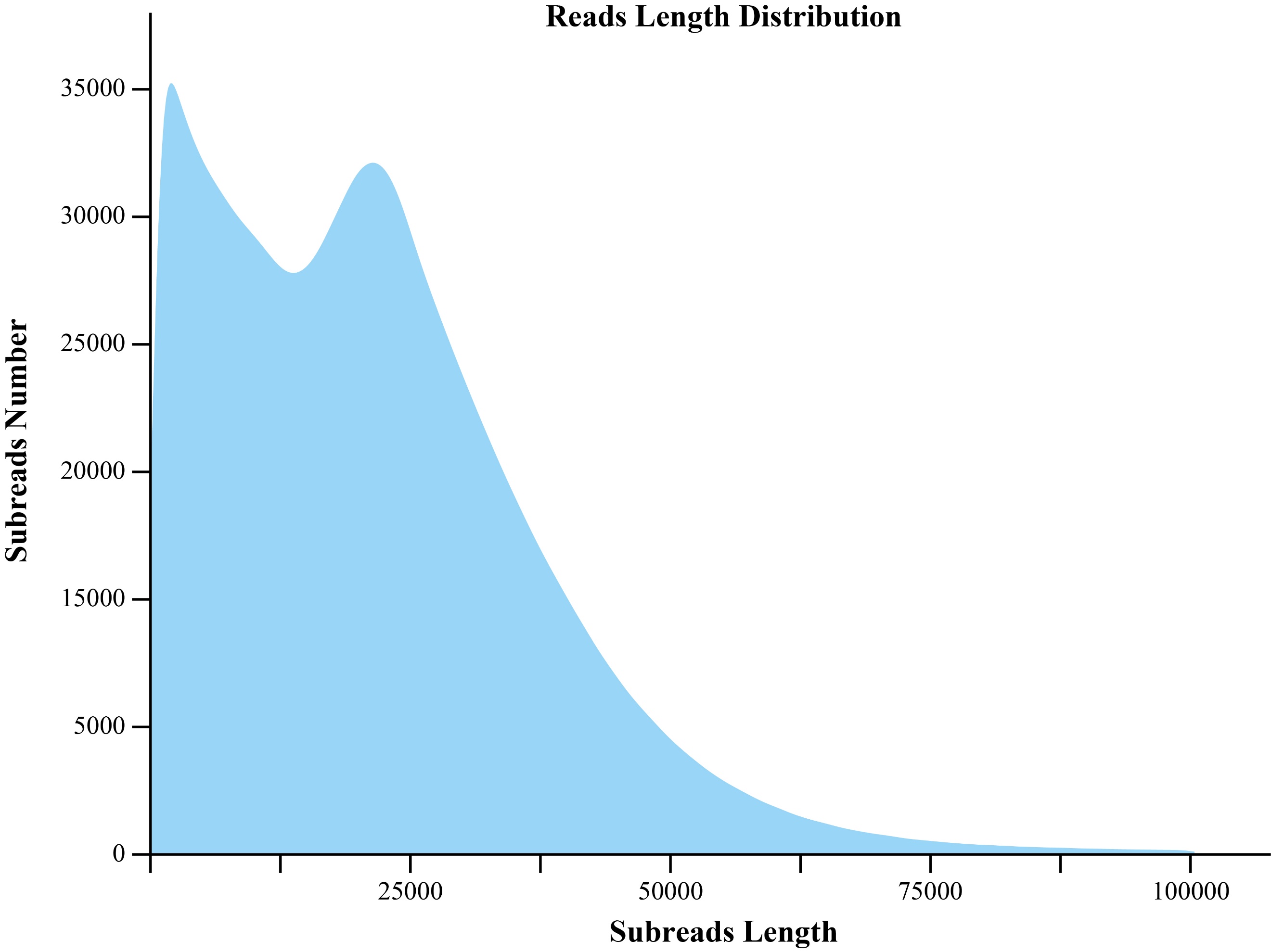
**

**Figure S1 The distribution of subreads length from genomic PacBio sequencing.**

**
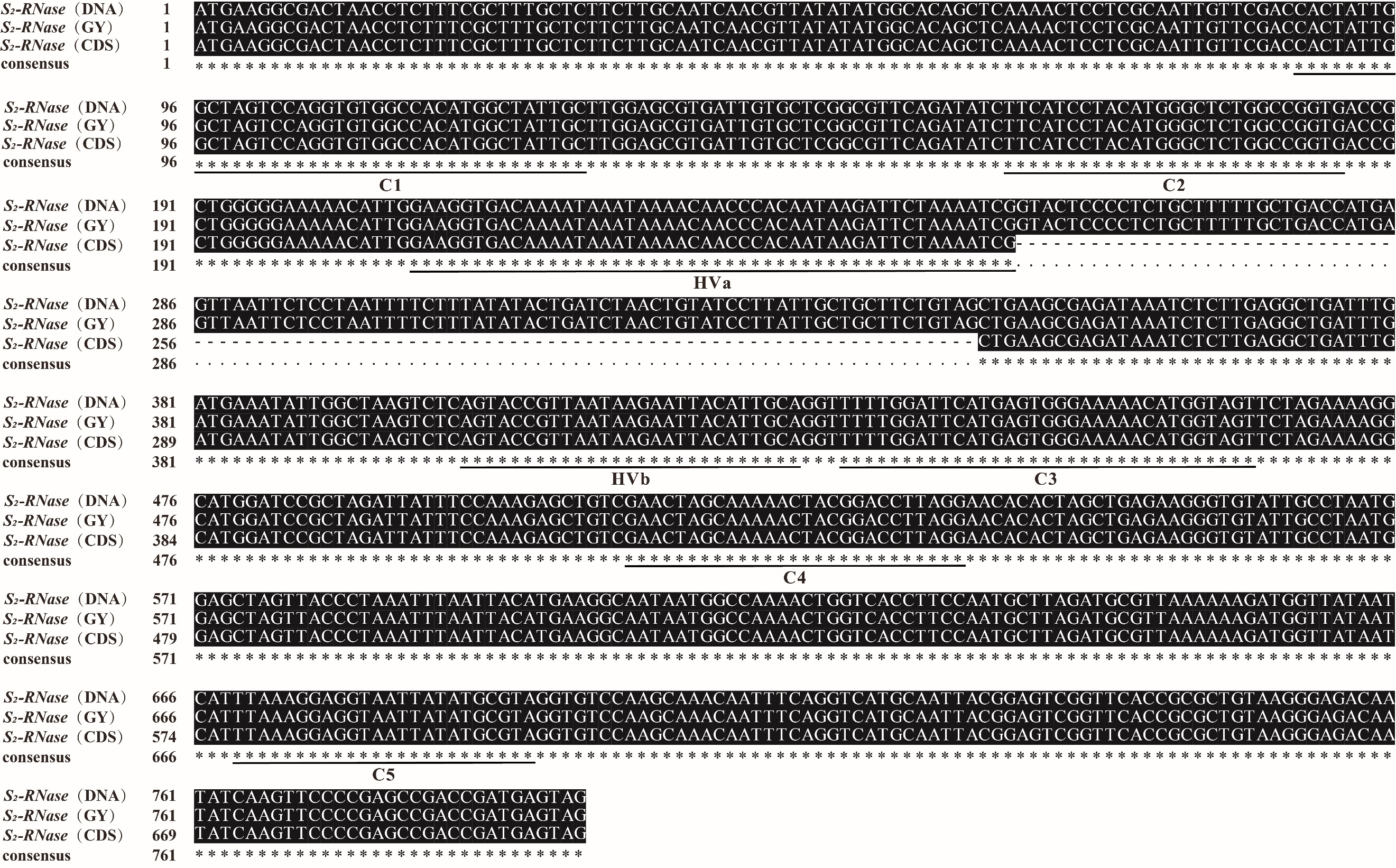
**

**Figure S2** **Nucleotide sequence alignment of *S_2_-RNase* between ‘ST’ and ‘GY’ pummelo.** The nucleotide sequence identities above 80% among *S_2_-RNase* are indicated by shaded boxes: *black indicates* 100% conservation; *grey indicates* ≥ 80% conservation; *dashes* represent gaps; *asterisks* indicate 100% conservation. The citrus *S-RNases* contain five conserved domains (C1-C5) and five hypervariable regions (HV1-HV5) ^29^.


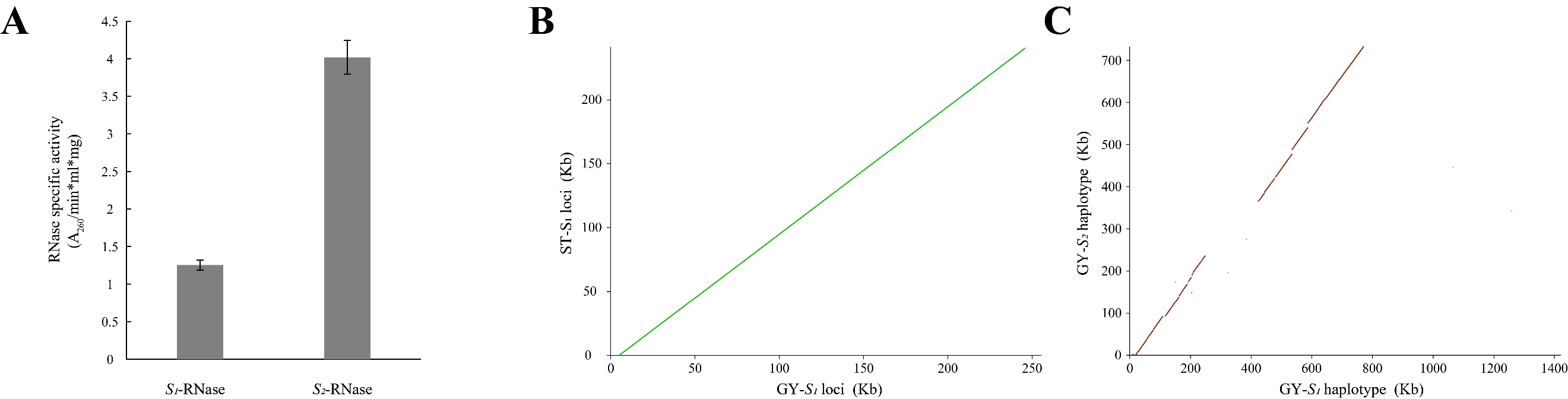


**Figure S3** **Structural variants analysis of *S* loci. A:** *S*-RNase activity analysis. The activity of *S_2_*-RNase was higher than *S_1_*-RNase. Experiments were repeated independently three times. **B:** Whole-sequence alignments of the *S*-locus region in *S_1_* haplotypes against the corresponding loci of ‘ST’ . The ST *S_1_* & *S_2_*-loci was obtained from publicly available databases (see Supplementary Table 3 for more detail). **C:** Whole-sequence synteny analysis of the ‘GY’ pummelo *S_1_* and *S_2_* haplotypes. GY-*S_1_* haplotype and GY-*S_2_* haplotype were obtained from phased assembled genome. Haplotype phasing was carried out using CANU (v2.1.1).


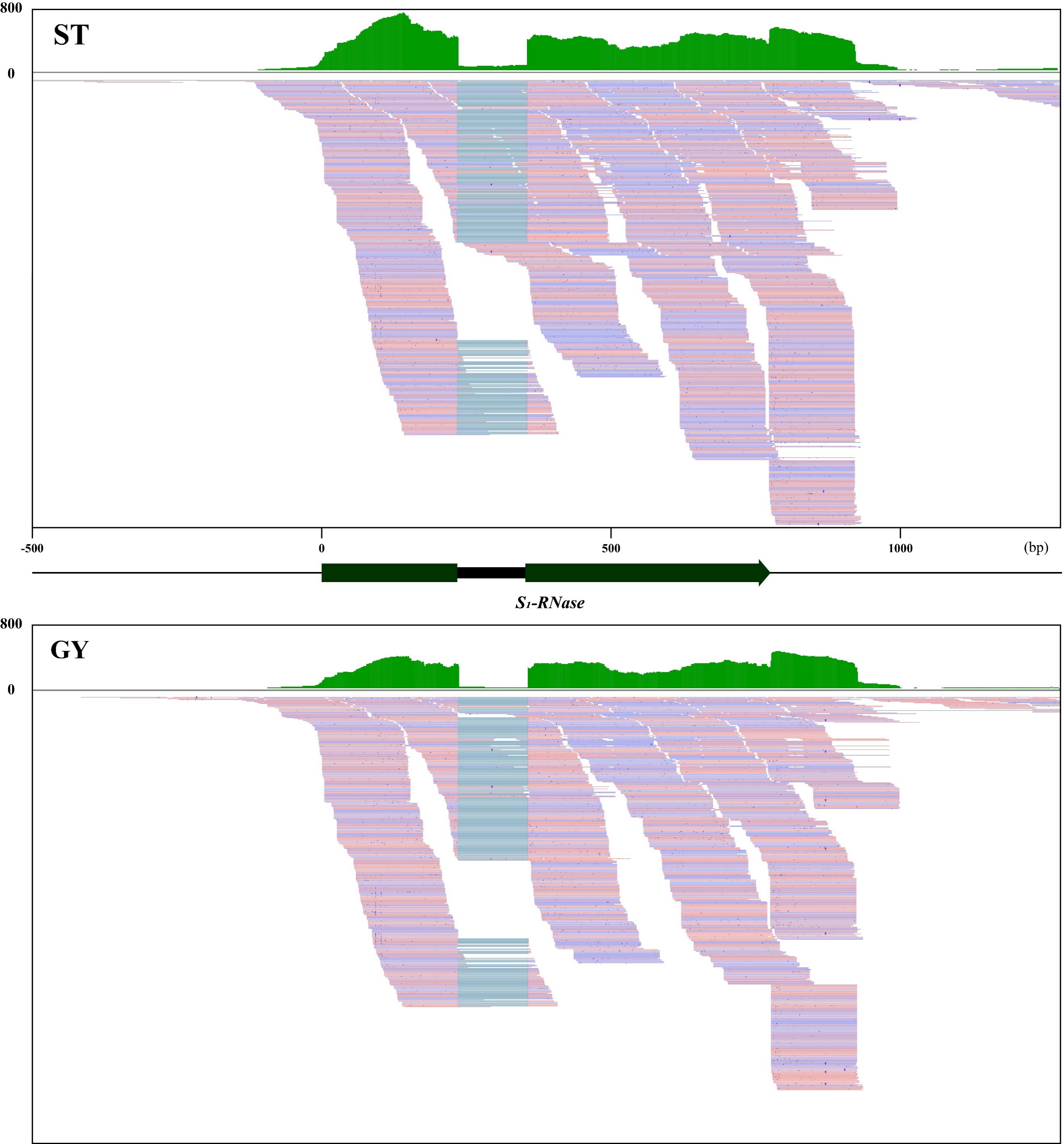


**Figure S4** **Integrative Genomics Viewer tracks displaying sequencing read clusters of *S_1_-RNase* gene from RNA-seq in styles of ‘ST’ and ‘GY’ pummelo.** The green bars depict the number of the reads mapped to the ‘GY’ assembled genome reference. There are clearly equivalent reads mapped to *S_1_-RNase* in styles of ‘ST’ and ‘GY’. Partial alignment of the RNA mapping is shown below, with pink and blue representing the different read strands.


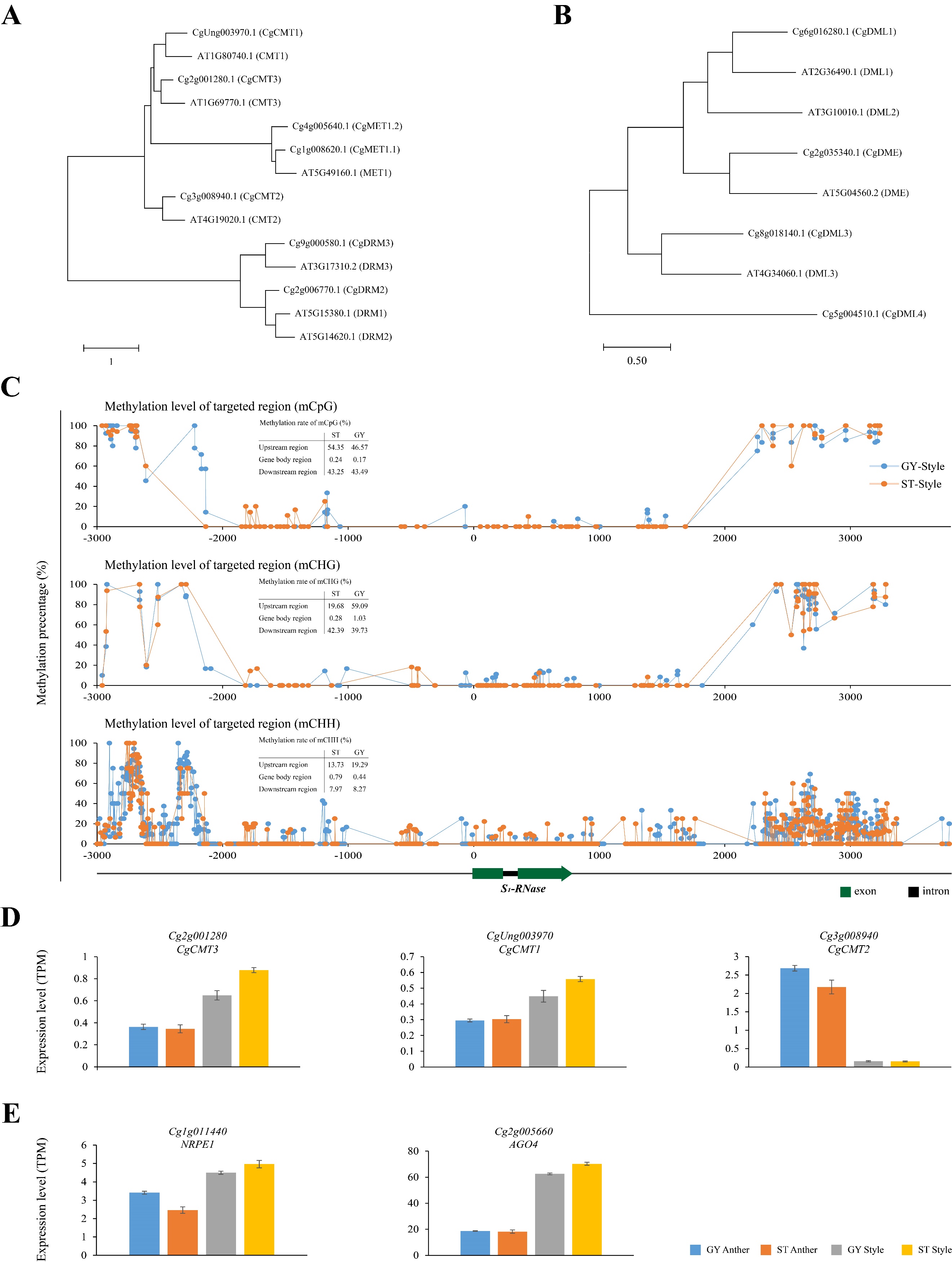


**Figure S5** **Methylation status of *S_1_-RNase* in styles of ‘ST’ and ‘GY’.** **A:** Phylogenetic analysis of DNA methyltransferase genes in HWB *C. maxima* genome. **B:** Phylogenetic analysis of DNA demethylase genes in HWB *C. maxima* genome. The unrooted dendo grams were generated using the Maximum Likelihood method in MEGA v10.0.5 based on the JTT model. **C:** Methylation levels at CpG (top row), CHG (middle row) and CHH (bottom row) sites in *S_1_-RNase* upstream (5’ flanking 3-kb regions), exons, intron and downstream (3’ flanking 3-kb regions) regions. Percentage in total mC is the number of mC/number of total C. **D:** Expression of DNA methyltransferase genes in anthers and styles between ‘ST’ & ‘GY’. Transcript levels of DNA methyltransferase genes in anther and style tissues. **E:** Transcript levels of two genes involved in RdDM. Error bars indicate means ± SE, *n* = 3. Percentage of methylation levels of *S* loci region were calculated by bismark (v0.22.2), ‘GY’ assembled genome as reference, due to the *S-RNase* on the *S*-locus of publicly pummelo *C. maxima* genome is identical to *S_6_-RNase*.


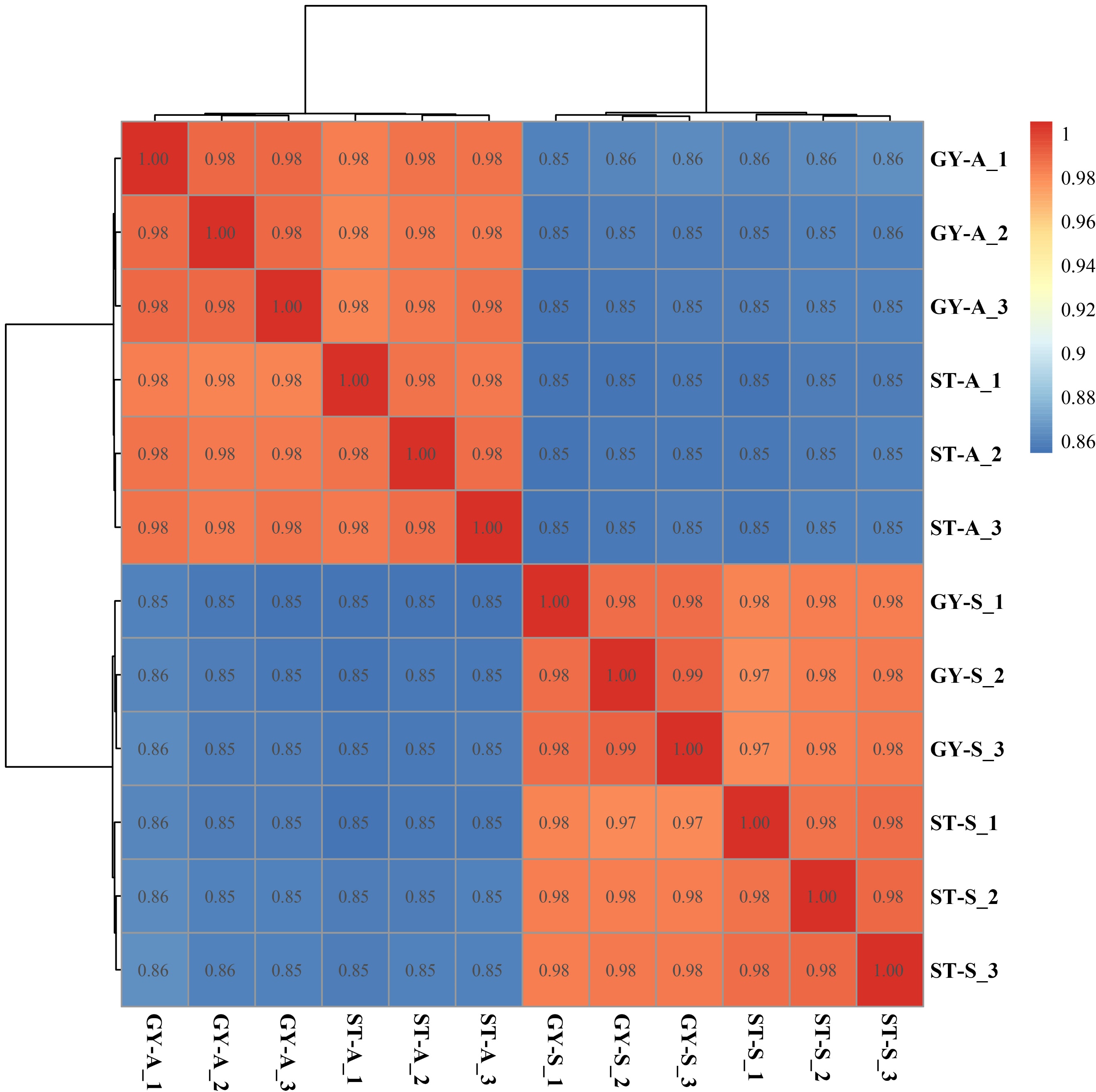


**Figure S6** **Clustering of 12 samples based on the Spearman rank correlation.** Sample name is designate as A-B_C, with A indicating the accession code (Supplementary Table 1), B indicates the tissue (“S” means style tissues; “A” represents Anther tissues), C indicates the repetition.


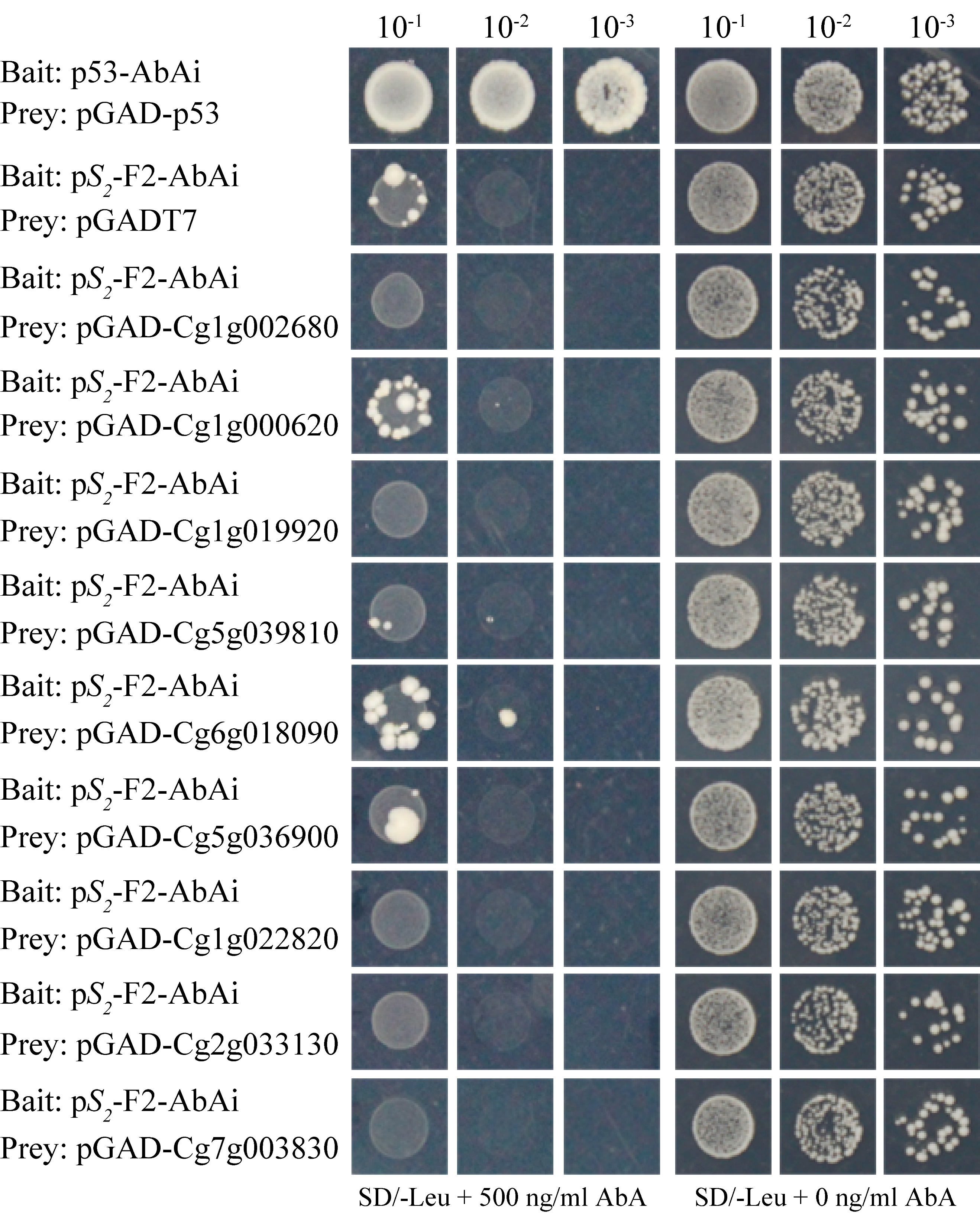


**Figure S7 Interactions between TF genes and the promoter of *S_2_-RNase*.** Interactions between TF genes and the promoter of *S_2_-RNase* were demonstrated using the yeast one-hybrid system. p53-AbAi+pGAD and p*S_2_*-F2+pGADT7 were expressed in yeast as positive and negative controls, respectively.

**Supplementary Tables**

**Supplementary Table 1.** Overview of the pummelo accessions used in the pollinations in this study.

| **No.** | **Accession name** | **Common name** | **Species** | **Scientific name** | **Harvest place of the samples** |
| --- | --- | --- | --- | --- | --- |
| 1 | ST | Shatian pummelo | Pummelo | *C. maxima* | Yulin City, Guangxi, China |
| 2 | GY | Guiyou NO.1 pummelo | Pummelo | *C. maxima* | Yulin City, Guangxi, China |

**Supplementary Table 2.** Detailed information relating to data from citrus style and anther RNA-seq libraries.

| **Species (*S*-genotype)** | **Sample name^a^** | **Tissue^b^** | **Number of read pairs** | **Clean bases (G)** | **Read length (bp)** | **GC (%)** | **CNR id in CNGB** |
| --- | --- | --- | --- | --- | --- | --- | --- |
| "Guiyou NO.1" pummelo (*S_1_S_2_*) | GY-A_1 | Anther from -1 DBA | 23698514 | 14.8 | 150 | 44.58 | CNR0369348 |
|  | GY-A_2 |  | 22922168 | 14.4 | 150 | 44.70 | CNR0369349 |
|  | GY-A_3 |  | 23682973 | 14.8 | 150 | 44.56 | CNR0369350 |
|  | GY-S_1 | Style from -1 DBA | 20241554 | 12.8 | 150 | 43.82 | CNR0369351 |
|  | GY-S_2 |  | 24235316 | 15.2 | 150 | 44.28 | CNR0369352 |
|  | GY-S_3 |  | 24281155 | 15.2 | 150 | 44.47 | CNR0369353 |
| "Shatian" pummelo (*S_1_S_2_*) | ST-A_1 | Anther from -1 DBA | 23671395 | 14.8 | 150 | 44.62 | CNR0369354 |
|  | ST-A_2 |  | 23658410 | 14.8 | 150 | 44.73 | CNR0369355 |
|  | ST-A_3 |  | 23324956 | 14.6 | 150 | 44.70 | CNR0369356 |
|  | ST-S_1 | Style from -1 DBA | 20738803 | 13 | 150 | 43.74 | CNR0369357 |
|  | ST-S_2 |  | 23950503 | 15 | 150 | 44.53 | CNR0369358 |
|  | ST-S_3 |  | 24232958 | 15.2 | 150 | 44.32 | CNR0369359 |

Data from 12 RNA-seq libraries of style and anther from two pummelos ("Shatian" and "Guiyou NO.1").

a: Sample name is designate as A-B-C, with A indicating the accession code (Supplementary Table 1), B indicates the tissue, C indicates the repetition.

b: -1 DBA represents 1 day before anthesis.

**Supplementary Table 3.** Detailed information relating to whole-sequence alignments of S loci.

| **No.** | **Accession name** | **Common name** | **Catalog** | **Scientific name** | **Source** | **BioProject** |
| --- | --- | --- | --- | --- | --- | --- |
| 1 | ST-*S_1_* loci | Shatian pummelo | Pummelo | *C.grandis* | Liang et al., 2020 | PRJNA573817 |
| 2 | ST-*S_2_* loci | Shatian pummelo | Pummelo | *C.grandis* | Liang et al., 2020 | PRJNA573818 |

**Supplementary Table 4.** List of primers used in this study.

| **Name** | **Direction** | **Primer sequence (5’ to 3’)** | **Purpose** |
| --- | --- | --- | --- |
| CgRNS1-SpeA_F | Forward | ATGAACATTACTTTCTTCCTCTACA | Specific amplification of *S_1_-RNase* |
| CgRNS1-SpeA_R | Reverse | GTTGATCTCCGTTCTTCGTG |  |
| CgRNS2-SpeA_F | Forward | GACTAACCTCTTTCGCTTTGC | Specific amplification of *S_2_-RNase* |
| CgRNS2-SpeA_R | Reverse | CGGATCCATGCCTTTTCTAG |  |
| CgRNS1-RTPCR_F | Forward | AAGGCCATCAGATTTCGTTC | RT-PCR of *S_1_-RNase* |
| CgRNS1-RTPCR_R | Reverse | CAGTGCTCATCCATTTCCATAC |  |
| CgRNS2-RTPCR_F | Forward | GGCCACATGGCTATTGCTTG | RT-PCR of *S_2_-RNase* |
| CgRNS2-RTPCR_R | Reverse | CCAATGTTTTTCCCCCAGCG |  |
| Actin_F | Forward | CCGACCGTATGAGCAAGGAAA | RT-PCR of *Actin* |
| Actin_R | Reverse | TTCCTGTGGACAATGGATGGA |  |
| CgHB40-QRT_F | Forward | CGAGTCTGAGGTGACAACGCT | QRT-PCR of CgHB40 |
| CgHB40-QRT_R | Reverse | GGTGAGTCCACGGTGTCCAT |  |
| CgHB40-Ful_F | Forward | ATGATCAGAGAAATGATCATAAC | gene clone and vector construction |
| CgHB40-Ful_R | Reverse | TTAATAGGTGTGGAGTAAGAGCCAC |  |
| pS2-1_F | Forward | ACAAGTCACACACACGCTCACG | promoter clone and vector construction |
| pS2-1_R | Reverse | ATCCTCATTGTTCAGCCTCCTT |  |
| pS2-2_F | Forward | CAGAGCATGGAGGAACGACA | promoter clone and vector construction |
| pS2-2_R | Reverse | TGCCTCATTTCATCCTTGGCA |  |
| pS2-3_F | Forward | TCACATTTACCTTTCGATCA | promoter clone and vector construction |
| pS2-3_R | Reverse | TTGGGAGAATGATACTTTCA |  |
| pS2-4_F | Forward | TGCATCCCAACCTGTAAGCA | promoter clone and vector construction |
| pS2-4_R | Reverse | GGATGCCAAAGGTAATGGTCC |  |
| pS2-5_F | Forward | ATGCCTTTCCATTTCGTCCA | promoter clone and vector construction |
| pS2-5_R | Reverse | CGAGGAGTTTTGAGCTGTGC |  |

**Supplementary Table 5.** Other genes identified at the GY *S_1_*-locus with functions predicted by the Non-redundant protein sequences (nr) databases.

| **Gene** | **Annotation** | **E value** | **Description** |
| --- | --- | --- | --- |
| S1_gene1 | transformation/transcription domain-associated protein | 0 | S1_Cg1g002040 |
| S1_gene2 | probable LRR receptor-like serine/threonine-protein kinase At3g47570 | 4.00E-76 | DN19064 |
| S1_gene3 | uncharacterized protein LOC107174676 | 2.00E-48 | DN12527 |
| S1_gene4 | F-box/kelch-repeat protein At3g23880-like | 0 | SLF1-1 |
| S1_gene5 | putative F-box protein At3g16210 | 0 | SLF1-2 |
| S1_gene6 | ribonuclease 1-like | 1.00E-45 | CgRNS1 |
| S1_gene7 | F-box/kelch-repeat protein At3g06240 | 0 | SLF1-3 |
| S1_gene8 | F-box/kelch-repeat protein At3g23880 | 0 | SLF1-4 |
| S1_gene9 | putative F-box protein At3g16210 | 0 | SLF1-5 |
| S1_gene10 | putative F-box protein At3g16210 | 0 | SLF1-6 |
| S1_gene11 | putative F-box protein At1g32420 | 0 | SLF1-7 |
| S1_gene12 | F-box/kelch-repeat protein At3g06240-like | 4.00E-170 | SLF1-8 |
| S1_gene13 | F-box/kelch-repeat protein At3g06240-like | 0 | SLF1-9 |
| S1_gene14 | calcium-dependent protein kinase 28-like isoform X3 | 0 | S1_Cg1g001860 |
| S1_gene15 | DNA-(apurinic or apyrimidinic site) lyase 2 isoform X1 | 0 | S1_Cg1g001850 |
| S1_gene16 | F-box/kelch-repeat protein At3g23880-like | 0 | SLF1-10 (Cg1g001840) |
| S1_gene17 | F-box protein At3g22700-like | 0 | SLF1-11 (Cg1g001830) |
| S1_gene18 | F-box protein CPR1-like | 0 | SLF1-12 (Cg1g001820) |
| S1_gene19 | F-box/kelch-repeat protein At3g23880-like | 1E-41 | S1_DN1593 |
| S1_gene20 | E3 ubiquitin-protein ligase Os03g0188200-like | 2E-97 | S1_Cg1g001800 |

**Supplementary Table 6.** Other genes identified at the GY *S_2_*-locus with functions predicted by the Non-redundant protein sequences (nr) databases.

| **Gene** | **Annotation** | **E value** | **Description** |
| --- | --- | --- | --- |
| S2_gene1 | transformation/transcription domain-associated protein | 0 | S2_Cg1g002040 |
| S2_gene2 | putative F-box protein At3g16210 | 0 | SLF2-1 |
| S2_gene3 | F-box/kelch-repeat protein At3g23880-like | 0 | SLF2-2 |
| S2_gene4 | putative F-box protein At3g16210 | 0 | SLF2-3 |
| S2_gene5 | putative F-box protein At3g16210 | 0 | SLF2-4 |
| S2_gene6 | F-box/kelch-repeat protein At3g23880 | 0 | SLF2-5 |
| S2_gene7 | ribonuclease 1-like | 3.00E-59 | CgRNS2 |
| S2_gene8 | F-box/kelch-repeat protein At3g06240 | 0 | SLF2-6 |
| S2_gene9 | putative F-box protein At1g32420 | 0 | SLF2-7 |
| S2_gene10 | F-box/kelch-repeat protein At3g06240-like | 3.00E-168 | SLF2-8 |
| S2_gene11 | F-box/kelch-repeat protein At3g06240-like | 0 | SLF2-9 |
| S2_gene12 | calcium-dependent protein kinase 28-like isoform X3 | 0 | S2_Cg1g001860 |
| S2_gene13 | DNA-(apurinic or apyrimidinic site) lyase 2 isoform X1 | 0 | S2_Cg1g001850 |
| S2_gene14 | F-box/kelch-repeat protein At3g23880-like | 0 | SLF2-10 (Cg1g001840) |
| S2_gene15 | F-box protein At3g22700-like | 0 | SLF2-11 (Cg1g001830) |
| S2_gene16 | F-box protein CPR1-like | 0 | SLF2-12 (Cg1g001820) |
| S2_gene17 | F-box/kelch-repeat protein At3g23880-like | 1E-41 | S2_DN1593 |
| S2_gene18 | E3 ubiquitin-protein ligase Os03g0188200-like | 2E-97 | S2_Cg1g001800 |

**Supplementary Table 7.** Expression level of genes at the *S_1_* and *S_2_*-locus in style tissues of GY pummelo.

| **gene** | **GY-A_1** | **GY-A_2** | **GY-A_3** | **GY-S_1** | **GY-S_2** | **GY-S_3** | **ST-A_1** | **ST-A_2** | **ST-A_3** | **ST-S_1** | **ST-S_2** | **ST-S_3** |
| --- | --- | --- | --- | --- | --- | --- | --- | --- | --- | --- | --- | --- |
| S1_Cg1g002060 | 308.2546 | 302.8153 | 293.0689 | 69.81863 | 109.4938 | 105.0618 | 287.5369 | 283.5858 | 269.3951 | 12.83938 | 24.4526 | 24.41016 |
| S1_Cg1g002050 | 0.394979 | 0.336303 | 0.439048 | 3.090203 | 6.592815 | 5.353225 | 0.328695 | 0.461769 | 0.474329 | 1.933471 | 2.493263 | 1.09397 |
| S1_Cg1g002040 | 1.23762 | 1.722304 | 1.925717 | 32.47775 | 40.69064 | 42.58513 | 0.470449 | 1.585187 | 2.153893 | 16.68713 | 27.55044 | 28.06999 |
| DN19064 | 0.112896 | 0.221827 | 0.366019 | 3.613368 | 4.522592 | 4.016521 | 0.117438 | 0.116459 | 0.059813 | 1.657922 | 1.187742 | 1.172579 |
| DN12527 | 0 | 0 | 0.038638 | 0 | 1.253218 | 1.695977 | 0 | 0.043028 | 0 | 0.408367 | 0.219417 | 0.216616 |
| SLF1-1 | 7.546612 | 8.805792 | 8.398152 | 0 | 0 | 0 | 7.796637 | 9.671183 | 10.6984 | 0 | 0 | 0 |
| SLF1-2 | 6.755464 | 7.822866 | 7.946988 | 0.274038 | 0.257246 | 0 | 8.603699 | 7.92254 | 9.961591 | 0.251474 | 0 | 0 |
| CgRNS1 | 0 | 0 | 0 | 402.7393 | 286.1909 | 295.2027 | 0 | 0.087214 | 0 | 267.7681 | 207.4701 | 219.7495 |
| SLF1-3 | 16.27581 | 15.96513 | 17.74527 | 0 | 0 | 0 | 14.80113 | 16.32017 | 15.77321 | 0 | 0 | 0 |
| SLF1-4 | 11.25218 | 11.12826 | 12.18335 | 0 | 0 | 0 | 15.76256 | 12.07157 | 12.79733 | 0 | 0 | 0 |
| SLF1-5 | 18.5749 | 19.21961 | 21.14969 | 0 | 0 | 0 | 22.61725 | 21.06936 | 22.2063 | 0 | 0 | 0 |
| SLF1-6 | 16.40873 | 18.72063 | 17.9313 | 0 | 0 | 0 | 19.69082 | 19.21463 | 20.53849 | 0 | 0 | 0 |
| SLF1-7 | 14.45115 | 13.83081 | 15.20642 | 0 | 0 | 0 | 16.11923 | 16.16444 | 15.44444 | 0 | 0 | 0 |
| SLF1-8 | 11.90757 | 10.79668 | 11.12203 | 0 | 0 | 0 | 14.1672 | 12.23217 | 12.93288 | 0 | 0 | 0.128829 |
| SLF1-9 | 8.682603 | 8.432625 | 8.594293 | 0 | 0 | 0 | 14.6059 | 10.61998 | 10.9614 | 0 | 0 | 0 |
| S1_Cg1g001860 | 27.2693 | 28.40869 | 30.01872 | 205.6682 | 222.3024 | 216.5129 | 20.68099 | 25.58943 | 29.88002 | 92.69019 | 122.5121 | 115.358 |
| S1_Cg1g001850 | 2.889106 | 2.794473 | 2.510671 | 28.12205 | 28.63594 | 27.85368 | 2.277241 | 2.42724 | 2.761519 | 12.97614 | 14.72763 | 13.53421 |
| S1_Cg1g001840 | 5.330352 | 6.085281 | 5.48618 | 0.263278 | 0 | 0 | 5.570469 | 6.516823 | 6.563327 | 0 | 0 | 0 |
| S1_Cg1g001830 | 13.49094 | 14.7188 | 15.12536 | 0 | 0 | 0 | 15.58466 | 18.18349 | 17.65879 | 0 | 0 | 0 |
| S1_Cg1g001810 | 3.100266 | 3.838225 | 3.618947 | 1.882365 | 2.019446 | 1.793473 | 5.820721 | 3.562118 | 4.673908 | 1.727372 | 1.1933 | 1.963443 |
| S1_DN1593 | 7.650042 | 9.552194 | 10.19515 | 0.526555 | 0 | 0 | 8.625243 | 9.418845 | 11.40084 | 0 | 0 | 0 |
| S1_Cg1g001800 | 2.430817 | 3.261829 | 2.800885 | 0 | 0 | 0 | 3.453713 | 2.629848 | 2.135972 | 0 | 0 | 0 |
| **gene** | **GY-A_1** | **GY-A_2** | **GY-A_3** | **GY-S_1** | **GY-S_2** | **GY-S_3** | **ST-A_1** | **ST-A_2** | **ST-A_3** | **ST-S_1** | **ST-S_2** | **ST-S_3** |
| S1_Cg1g001790 | 7.177943 | 6.809753 | 7.162662 | 0 | 0 | 0 | 8.940837 | 7.59514 | 8.193434 | 0 | 0 | 0 |
| S2_Cg1g002060 | 282.7269 | 281.8945 | 268.0511 | 35.18637 | 61.63901 | 60.9781 | 259.0102 | 264.9944 | 246.4181 | 6.737498 | 17.89548 | 17.26243 |
| S2_Cg1g002050 | 0.526638 | 0.517389 | 0.707355 | 0.842783 | 3.955689 | 2.408951 | 0.493043 | 0.434607 | 0.697542 | 1.417879 | 1.80069 | 2.734926 |
| S2_Cg1g002040 | 1.947611 | 2.226334 | 2.614921 | 37.82153 | 50.18756 | 50.46944 | 1.246943 | 2.558372 | 3.25918 | 22.36456 | 36.10719 | 37.32564 |
| SLF2-1 | 15.4117 | 13.6774 | 13.89533 | 0 | 0 | 0 | 14.42856 | 14.22878 | 15.37787 | 0 | 0 | 0 |
| SLF2-2 | 15.7874 | 15.7093 | 15.84466 | 0 | 0.253788 | 0 | 17.74057 | 15.97194 | 17.3193 | 0 | 0.133302 | 0 |
| SLF2-3 | 12.82956 | 11.11847 | 14.49914 | 0 | 0 | 0 | 13.6079 | 16.35454 | 17.76085 | 0 | 0 | 0 |
| SLF2-4 | 37.60484 | 36.3404 | 37.42019 | 0 | 0 | 0 | 41.56925 | 40.6149 | 43.45674 | 0 | 0 | 0 |
| SLF2-5 | 17.09398 | 16.81846 | 16.92877 | 0 | 0 | 0 | 23.97913 | 18.178 | 18.8056 | 0 | 0 | 0 |
| CgRNS2 | 0 | 0 | 0 | 4.748038 | 5.267461 | 9.047639 | 0 | 0.041735 | 0 | 439.0755 | 396.4931 | 398.3649 |
| SLF2-6 | 10.42437 | 10.87831 | 12.08181 | 0 | 0 | 0 | 11.28477 | 11.62802 | 13.21271 | 0 | 0.131186 | 0 |
| SLF2-7 | 19.35375 | 19.55429 | 20.47543 | 0 | 0 | 0 | 24.47618 | 20.37722 | 22.52119 | 0 | 0.131534 | 0 |
| SLF2-8 | 3.454858 | 3.491157 | 4.411803 | 0 | 0 | 0 | 3.568181 | 4.200296 | 3.713117 | 0 | 0 | 0 |
| SLF2-9 | 19.05211 | 18.81499 | 19.69334 | 0 | 0 | 0 | 23.92167 | 21.87972 | 23.89428 | 0 | 0 | 0 |
| S2_Cg1g001860 | 26.84525 | 27.91198 | 30.01872 | 156.4261 | 160.5608 | 159.4835 | 19.5443 | 27.01948 | 30.00099 | 106.4221 | 129.7187 | 123.9972 |
| S2_Cg1g001850 | 2.442337 | 2.54575 | 2.483081 | 14.61711 | 14.91455 | 15.74339 | 2.401173 | 2.872746 | 3.266482 | 13.19484 | 15.11932 | 12.52881 |
| S2_Cg1g001840 | 6.070679 | 6.303478 | 5.371884 | 0 | 0 | 0 | 6.058206 | 6.822299 | 6.406435 | 0.1208 | 0 | 0 |
| S2_Cg1g001830 | 13.92381 | 12.40348 | 15.30357 | 0 | 0 | 0 | 15.68472 | 17.19121 | 17.73523 | 0 | 0 | 0 |
| S2_Cg1g001810 | 4.058072 | 4.581107 | 5.043178 | 1.882365 | 1.262154 | 1.793473 | 6.528646 | 5.772191 | 4.967696 | 1.727372 | 0.662944 | 1.832547 |
| S2_DN1593 | 8.439724 | 8.485452 | 10.69805 | 0 | 0 | 0 | 7.649769 | 9.622496 | 12.44679 | 0 | 0 | 0.25631 |
| S2_Cg1g001800 | 2.371529 | 3.087088 | 2.691046 | 0 | 0 | 0 | 3.268693 | 2.201733 | 2.57573 | 0 | 0 | 0 |
| S2_Cg1g001790 | 8.441014 | 8.353297 | 8.732169 | 0 | 0 | 0 | 9.966309 | 9.946773 | 9.499161 | 0 | 0 | 0 |

TPM values of genes in GY *S*-loci were calculated with the reads mapped to the assembled genome reference.

**Supplementary Table 8.** Summary informations of DNA methylation in style tissues of ST and GY pummelo.

|  | **GY** | **ST** |
| --- | --- | --- |
| total analysed C's | 884850507 | 896577493 |
| methylated CpG | 65570342 | 69965967 |
| unmethylated CpG | 39217093 | 39924984 |
| methylated CHG | 60290343 | 63807010 |
| unmethylated CHG | 77786168 | 78723454 |
| methylated CHH | 45037506 | 47126729 |
| unmethylated CHH | 596949055 | 597029349 |
| methylated CN | 17333 | 19113 |
| unmethylated CN | 217399 | 220704 |
| total methylated C's | 170915524 | 180918819 |
| total C's on genome | 101563118 | 101563118 |
| detected C's | 885085239 | 896817310 |
| detected rate | 8.714632402 | 8.830147475 |
| **#methylation rate of different context agaist total methylated contexts** | | |
| mCpG | 0.625746226 | 0.636685426 |
| mCHG | 0.436644456 | 0.447672787 |
| mCHH | 0.070153347 | 0.07316042 |
| mCN | 0.073841658 | 0.07969827 |
| mC | 0.193157514 | 0.201788268 |
| **#methylation rate in different context agaist total mCs** | | |
| CpG | 0.383641816 | 0.386725756 |
| CHG | 0.352749368 | 0.3526831 |
| CHH | 0.263507404 | 0.2604855 |
| Mapping efficiency | 40.52% | 39.81% |

**Supplementary Table 9.** Mapping summary for WGBS reads.

|  | **GY** | **ST** |
| --- | --- | --- |
| Total Reads | 121901830 | 124709378 |
| Aligned Reads | 49390114 | 49644636 |
| Unaligned Reads | 14847796 | 1577552 |
| Ambiguously Aligned Reads | 57663920 | 59289190 |
| No Genomic Sequence | 28 | 35 |
| Mapping rate | 0.878198744 | 0.873501478 |
| mean reads coverage | 16.58 | 16.60 |
| CNR id in CNGB | CNR0369360 | CNR0369361 |
