# Supplementary figures and images for: Downregulated expression of S2-RNase attenuates self-incompatibility in “Guiyou No. 1” pummelo
Source: Hortic Res. 2021 Sep 1;8:199. doi: 10.1038/s41438-021-00634-8 (PMC8408199; doi:10.1038/s41438-021-00634-8)

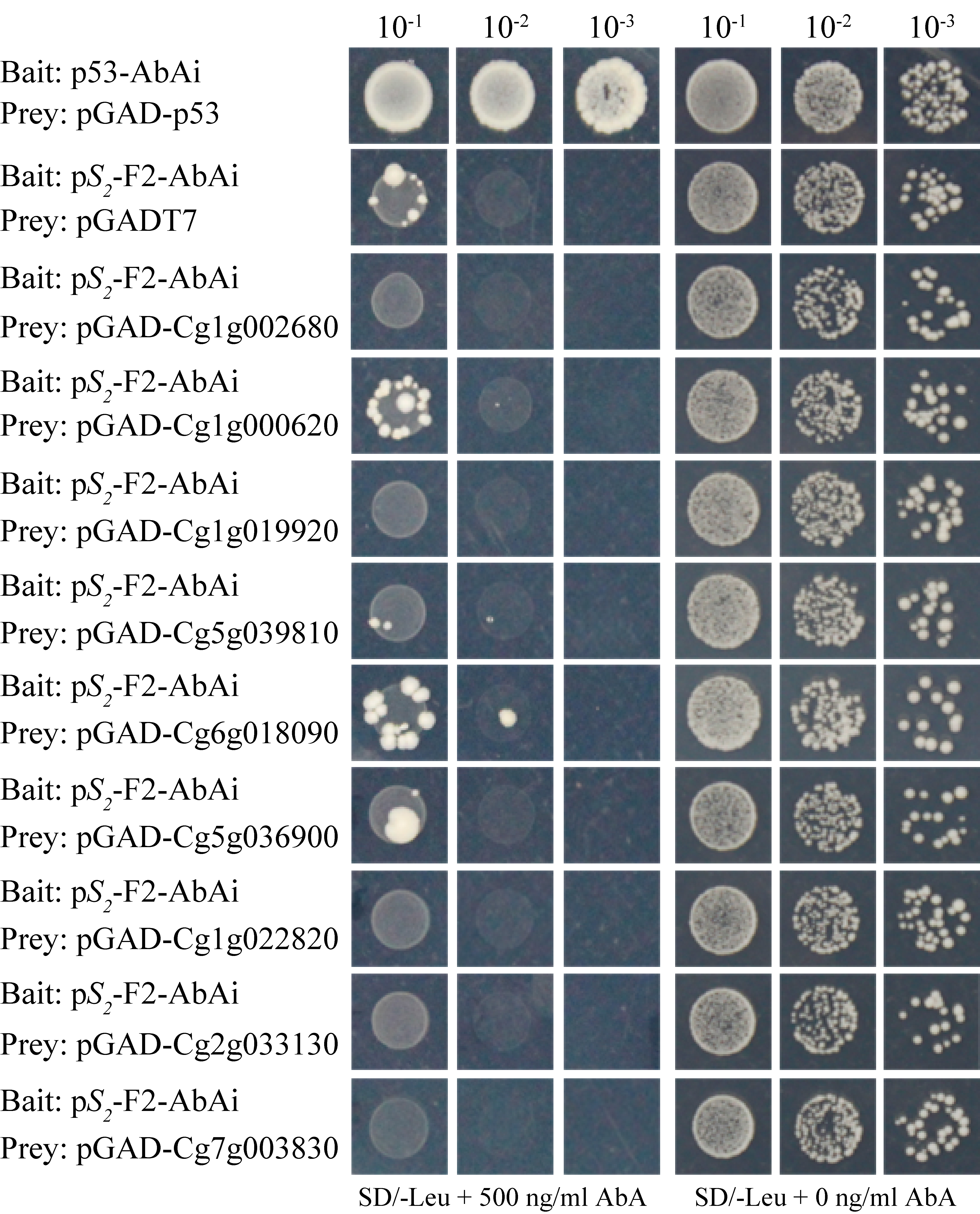

Supplement: Supplementary file 2 — Supplementary Figure 1 [file 41438_2021_634_MOESM2_ESM.jpg]

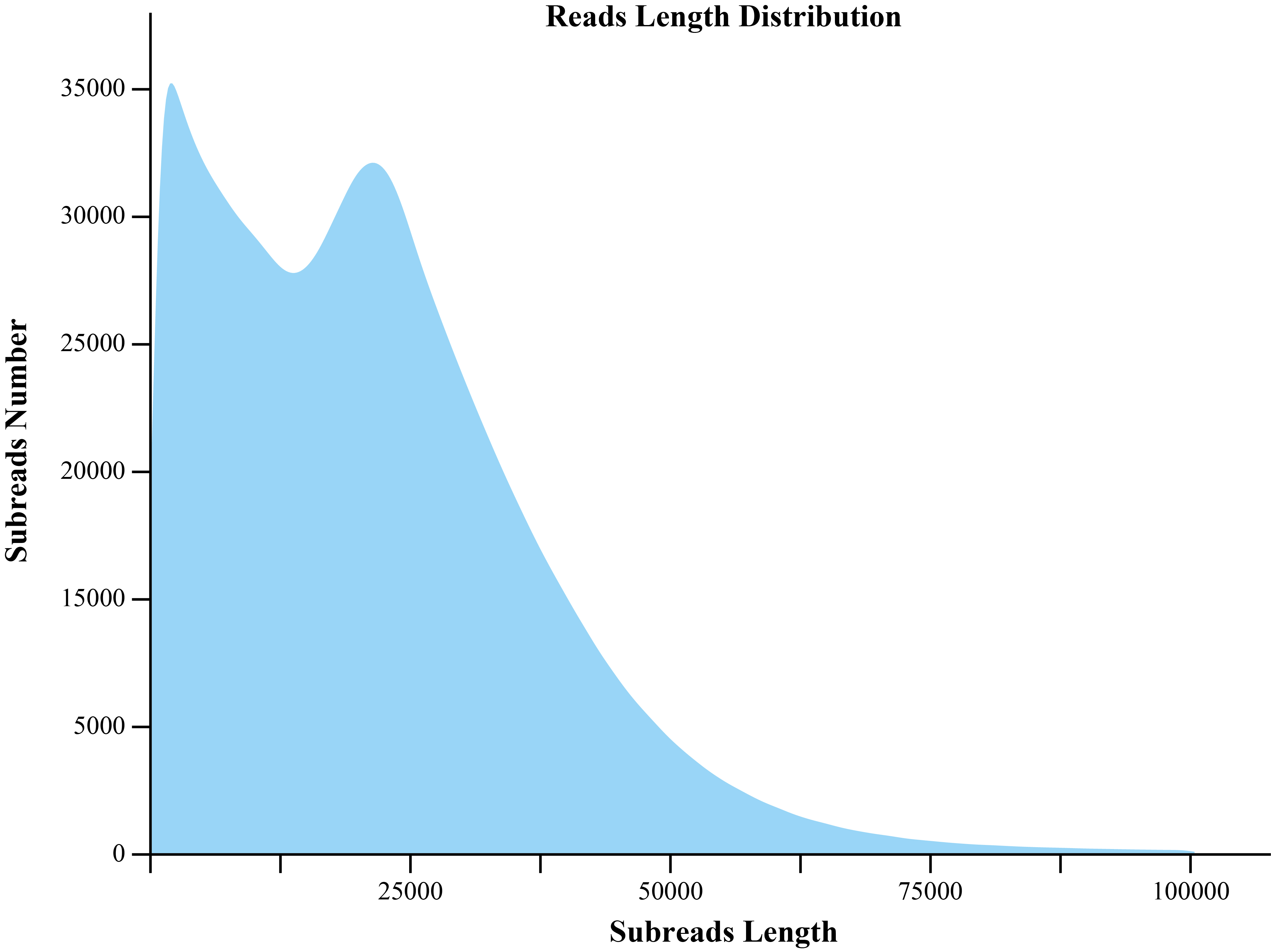

Supplement: Supplementary file 3 — Supplementary Figure 2 [file 41438_2021_634_MOESM3_ESM.jpg]

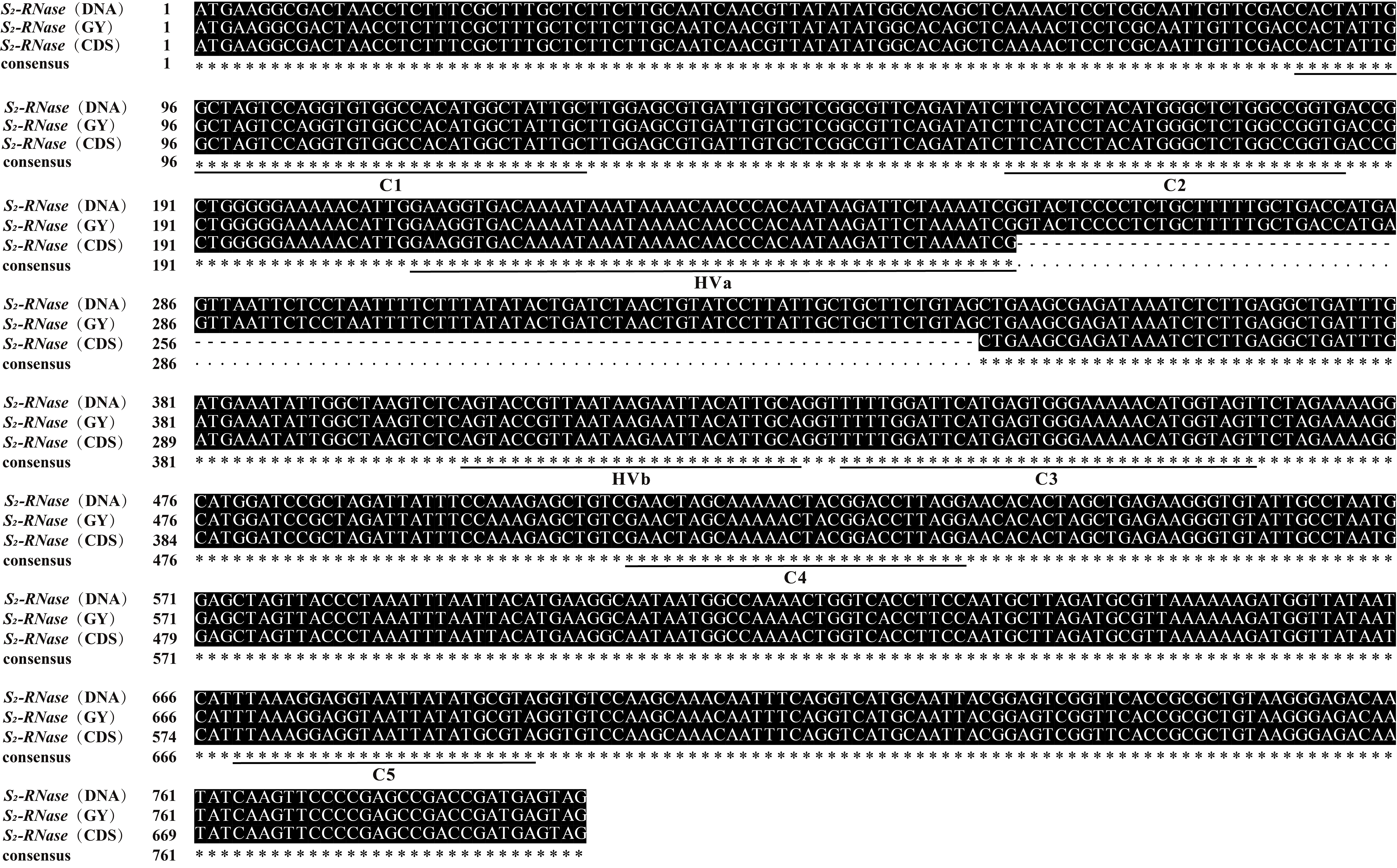

Supplement: Supplementary file 4 — Supplementary Figure 3 [file 41438_2021_634_MOESM4_ESM.jpg]

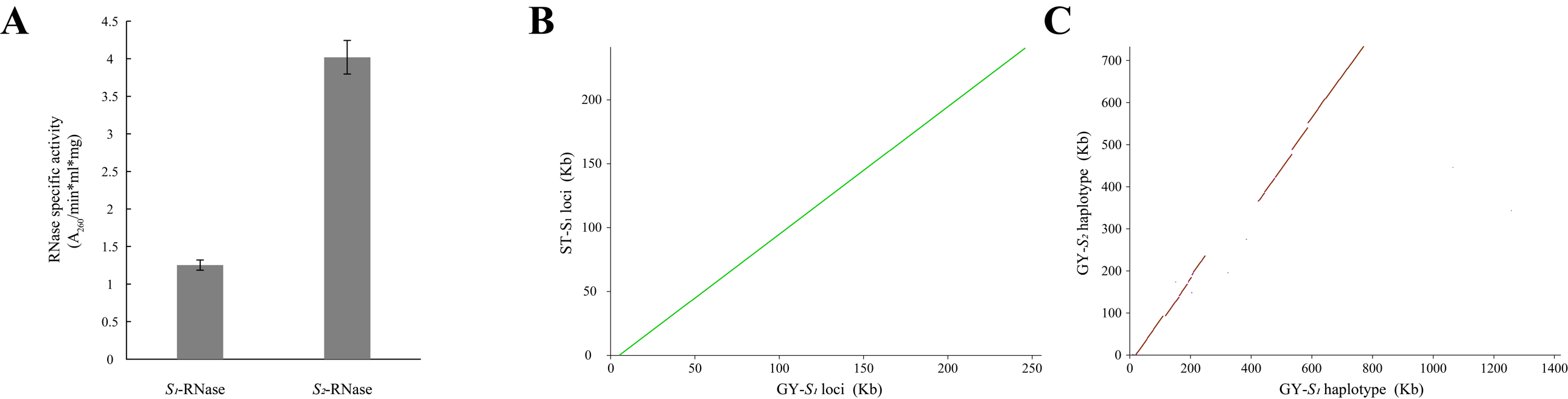

Supplement: Supplementary file 5 — Supplementary Figure 4 [file 41438_2021_634_MOESM5_ESM.jpg]

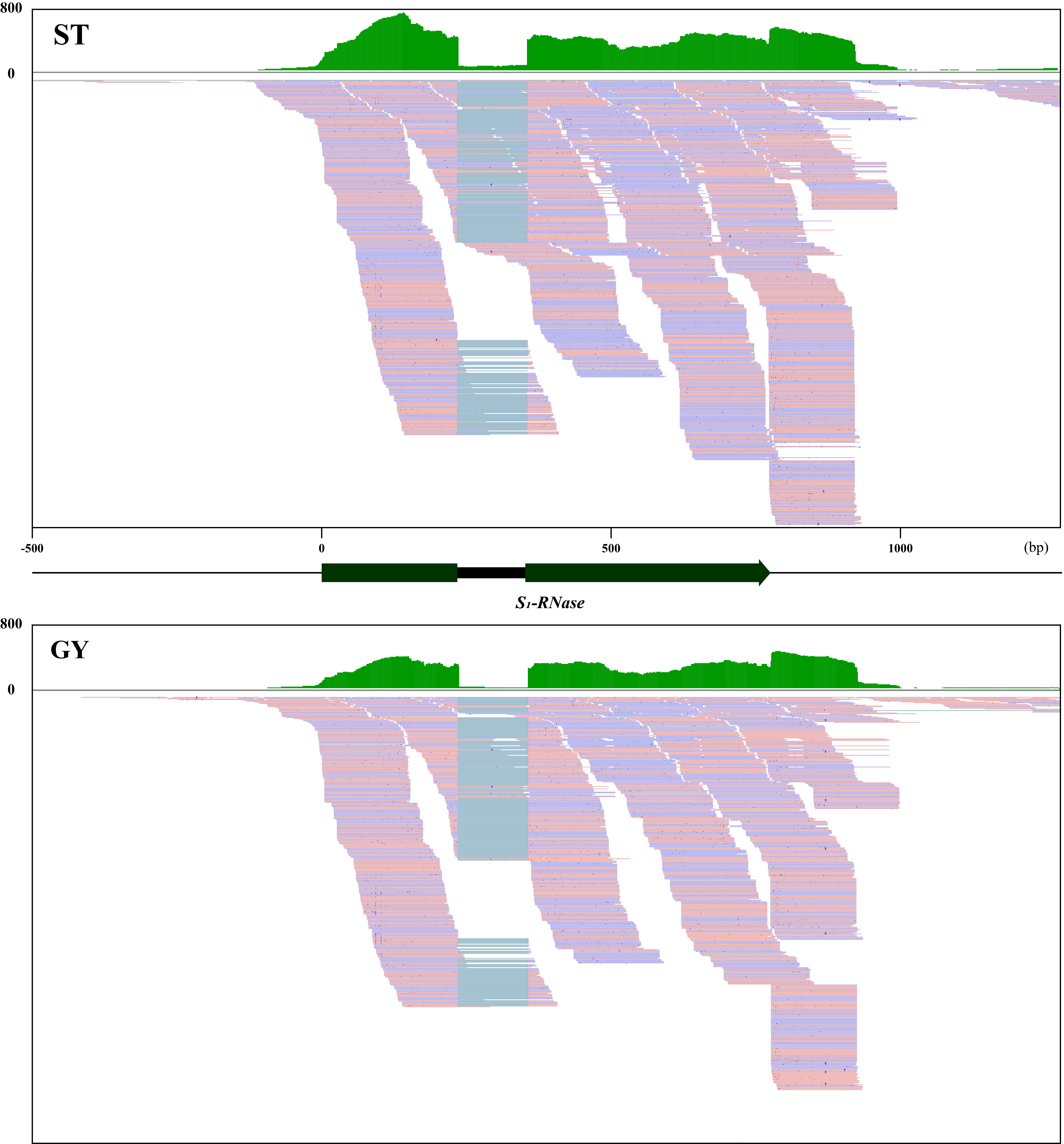

Supplement: Supplementary file 6 — Supplementary Figure 5 [file 41438_2021_634_MOESM6_ESM.jpg]

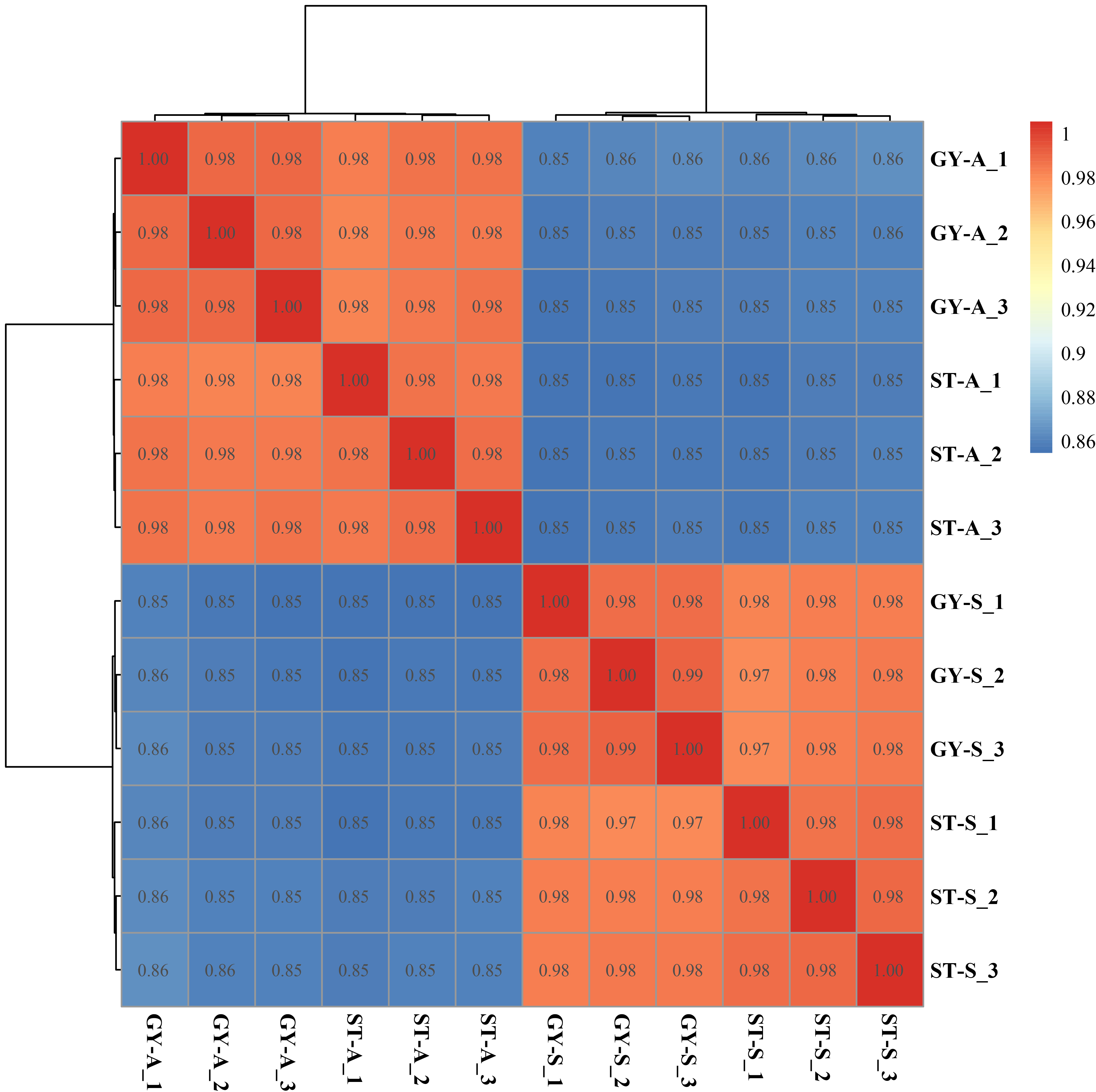

Supplement: Supplementary file 8 — Supplementary Figure 7 [file 41438_2021_634_MOESM8_ESM.jpg]
